# Phytochemical Compositions of Some Red Sea Halophyte Plants with Antioxidant and Anticancer Potentials

**DOI:** 10.3390/molecules27113415

**Published:** 2022-05-25

**Authors:** Usama W. Hawas, Lamia T. Abou El-Kassem, Fekri M. Shaher, Radwan Al-Farawati, Mohamed Ghandourah

**Affiliations:** 1 Marine Chemistry Department, Faculty of Marine Sciences, King Abdulaziz University, Jeddah 21589, Saudi Arabia; rfarawati@kau.edu.sa (R.A.-F.); mghandourah@kau.edu.sa (M.G.); 2 Department of Chemistry, College of Science and Art, King Abdulaziz University, Jeddah 22254, Saudi Arabia; lgadalmula@kau.edu.sa; 3 Marine Chemistry Department, Faculty of Marine Sciences and Environment, Hodeidah University, Al-Hudaydah, Yemen; fikry1978@yahoo.com

**Keywords:** halophytes, fatty acids, amino acids, phenolic, cytotoxicity, antioxidant

## Abstract

The aim of this study was to determine the compositions of carbohydrates, phenolic compounds, fatty acids (FAs), and amino acids (AAs) of four Rea Sea halophytes: *Anabasis ehrenbergii*, *Suaeda aegyptiaca*, *Suaeda monoica*, and *Zygophyllum album*. The results showed that *S. aegyptiaca* and *S. monoica* were rich in gallic acid with 41.72 and 47.48 mg/g, respectively, while *A. ehrenbergii* was rich in naringenin with 11.88 mg/g. The polysaccharides of the four species were mainly composed of galactose (54.74%) in *A. ehrenbergii*, mannose (44.15%) in *S. aegyptiaca*, glucose and ribose (33 and 26%, respectively) in *S. monoica*, and arabinose and glucose (36.67 and 31.52%, respectively) in *Z. album*. Glutamic acid and aspartic acid were the major AAs in all halophyte species with 50–63% and 10–22% of the total AAs, respectively. The proportion of unsaturated fatty acids (UFA) of the four species was 42.18–55.33%, comprised mainly of linolenic acid (15.54–28.63%) and oleic acid (5.68–22.05%), while palmitic acid (23.94–49.49%) was the most abundant saturated fatty acid (SFA). Phytol and 9,19-cyclolanost-24-en-3β–ol represented the major unsaponifiable matter (USM) constituents of *S. monoica* and *A. ehrenbergii* with proportions 42.44 and 44.11%, respectively. The phenolic fraction of *S. aegyptiaca* and *S. monoica* demonstrated noteworthy antioxidant activity with IC_50_ values of 9.0 and 8.0 μg/mL, respectively, while the FAs fraction of *Z. album* exhibited potent cytotoxic activity against Huh-7, A-549, and Caco-2 cancer cell lines with IC_50_ values of 7.4, 10.8, and 11.8 μg/mL, respectively. Our results indicate that these plants may be considered a source of naturally occurring compounds with antioxidant and anticancer effects that could be suitable for future applications.

## 1. Introduction

Halophytes are salt-tolerant plants that grow in a wide variety of saline habitats such as mangrove swamps, marshes, seashores, and saline semi-deserts [1]. The plants exhibit different strategies that adapt to osmotic, ionic, and oxidative stresses including delays in germination or growth, exclusion of salts in the root zone, excretion of salts via glands or hairs, controls and compartmentalization of ion uptake as well as an effective antioxidant system to reduce oxidative stress [2]. The antioxidant system is responsible for maintaining the level of reactive oxygen species (ROS) which is important for metabolic pathways [3]. A large number of halophytes are precious natural resources and have potential economic value as food, animal feed, biofuel feedstock, and coastal protection [4]. Besides, it has been discovered that some halophytes can grow on toxic metal soils, providing a novel way to treat environmental pollution [5].

Saudi Arabia is estimated to contain over 100 halophyte species divided into 33 families. Among these species, *Anabasis ehrenbergii*, *Suaeda aegyptiaca*, and *Suaeda monoica* (family: Chenopodiaceae), and *Zygophyllum album* (family: Zygophyllaceae) are important for their economic and therapeutic properties, mostly as ornamentals, folk remedies, and sources of food and fibers. *S. monoica* is mainly used in traditional medicine for the treatment of rheumatism, paralysis, asthma, snake-bites, skin disease, and ulcer as well as hepatitis [6,7]. Previous phytochemical studies of this plant have shown that it contained triterpenoids, phenolic compounds, amino acids, and fatty acids. Different extracts and some metabolites of the plant have been shown to possess in vitro cell proliferative, antioxidative, anti-apoptotic, hepatoprotective, and antimicrobial activities [6,8,9,10]. *S. aegyptiaca* is another species found around salt marshes and native to North Africa and Arabia. It is used traditionally for stomach pain, wound healing, and skin infection. However, this species is noted for its antidiabetic and antioxidant properties with limited studies on its chemical composition [11,12,13]. Likewise, there are no phytochemical data on *A. ehrenbergii*, however, its *Anabasis* genus is described in ethnomedical reports mainly for the treatment of diabetes, digestive tract disorders, rheumatism, and poison antidote [14,15,16]. *Z. album* commonly known as “Bougriba” is one of the most popular herb drugs widely distributed in arid regions. This plant is described in several ethnomedical surveys as antispasmodic, antirheumatic, and anti-eczema [17], and used as a diuretic, antihistaminic, and anaesthetic [18]. Furthermore, its crude extract and organic fractions exhibited considerable anti-inflammatory, antihyperlipidemic cytotoxic potential, as well as could reduce hepatotoxicity and nephrotoxicity [19,20]. A number of secondary metabolites flavonoids, triterpenoids, and fatty acids have been recently reported from this species [21,22].

Currently, an increasing number of halophyte species are being reported from various parts of the world. However, phytochemical analyses of these plants remain unexplored. Therefore, in the present study, four halophytes widely found along Red Sea shorelines: *A. ehrenbergii*, *S. aegyptiaca*, *S. monoica* and *Z. album* were assessed. These halophytes were investigated for their detailed compositions of fatty acids and other lipids (such as hydrocarbons and sterols), polysaccharides, amino acids, and phenolic compounds (Supplementary Material Figures S1–S20). The MeOH extracts and contents of polyphenol and lipoids were evaluated for their antioxidant activity using DPPH free radical scavenging method and cytotoxicity using the MTT assay.

## 2. Results and Discussion

### 2.1. Monosaccharide Compositions

The GC-MS analysis of the four halophytes polysaccharides after hydrolysis and derivatization to their volatile states revealed the presence of different carbohydrate moieties in varied proportions (Table 1). Analysis of monosaccharide compositions of the polysaccharide fractions showed that they consisted primarily of glucose, galactose, arabinose, mannose, and xylose, which accounted for the majority of monosaccharides present. Small amounts of rhamnose, galacturonic acid, glucuronic acid, and fucose were also detected in the fractions. The results also indicated that monosaccharide compositions of *A. ehrenbergii* consisted primarily of galactose and glucose with maximum values of 54.74 and 14.79%, respectively, while arabinose and glucose formed the backbone of the polysaccharides in *Z. album* with the highest percentages of 36.67 and 31.52% compared with the other monosaccharide compositions. Moreover, the polysaccharides in *S. monoica* were mainly comprised of glucose, ribose, and xylose with the majority of 33.0, 26.0, and 10.7%, respectively. In comparison, *S. aegyptiaca* was constituted mainly of mannose with the highest composition (44.15%) along with small molar ratios of 16.3, 9.63, 8.79, and 3.35% of glucose, galactose, ribose, and rhamnose, respectively. These results were different from previous reports on other *Saueda* species [23,24]. Besides the genetic variations in salt tolerance, the reasons may also be closely related to the growth environment of the plants as well as the extraction and purification methods.

### 2.2. Phenolic Compositions

Halophytes synthesized phenolic compounds in high amounts in order to survive under stressful conditions, particularly high levels of salinity [25]. The result in Table 2 showed that a total of 17 phenolic compounds corresponding to 6 flavonoids, 8 phenolic acids, phenolic ester, phenolic diol, and phenolic aldehyde were detected in the investigated phenolic contents of the halophytes with different concentrations. The highest total phenolic was found in *A. ehrenbergii* with 16 compounds, followed by *S. monoica* (15 compounds), *S. aegyptiaca* (12 compounds), and *Z. album* (11 compounds). Among these species, *S. aegyptiaca* and *S. monoica* were noteworthy for their extremely high gallic acid content with 41.72 and 47.48 mg/g, respectively, which was reported as an abundant phenolic component in other halophyte plants [26]. Ellagic acid was predominant in *A. ehrenbergii* with a value of 9.96 mg/g, while a high content of chlorogenic acid was found in *S. monoica* and *A. ehrenbergii* with values of 7.2 and 5.07 mg/g, respectively. The phenolic acids were presented in *Z. album* in very small amounts compared to the significant amounts of flavonoids naringenin and rutin (quercetin-3-*O*-rutinoside) with values of 6.42 and 5.17 mg/g, respectively. These flavonoids are also predominant in *A. ehrenbergii* with values of 11.88 and 4.79 mg/g, respectively, where the flavonoid metabolites are considered a chemotaxonomic marker in plants. However, hesperetin (flavonone), quercetin, and Kaempferol (flavonol) were in very few proportions in all investigated halophyte species. The phenolic patterns of the investigated halophytic species are consistent with those of other halophyte species [27]. Additionally, previous research conducted on the phenolic composition of non-halophyte *S. monoica* indicated the presence of fewer flavonoids and phenolic acids with lower concentrations in comparison with the same halophyte *Suaeda* species [10]. This could be attributed to the salinity stress condition under which the halophyte species grow which increases the synthesis of non-enzymatic molecules with antioxidant properties in order to decrease the production of reactive oxygen species (ROS) [3].

### 2.3. Amino Acid Compositions

The results for the protein content (%) and amino acid (AAs) composition (µg/g DW) of the four halophyte species are listed in Table 3. The protein content of the halophyte species ranged from 8.28% (*S. monoica*) to 5.72% (*Z. album*). These results are in agreement with a previous study for both *S. monoica* and *Z. album* by Ahmed and Lotfy, 2015 [28]. The high abundance of essential amino acids (EAA) was detected in *A. ehrenbergii* (230.68 µg/g)*,* comprised mainly of phenylalanine, leucine, methionine, and lysine with values of 77.13, 47.92, 39.15, and 24.96 µg/g, respectively. However, the ratio of EAA to non-EAA in all investigated species is low but *A. ehrenbergii* has the highest ratio (1:0.344). The highest amount of non-EAA was found in *Z. album* (1108.34 µg/g), represented 85.34% of total AAs, and comprised mainly of glutamic acid and aspartic acid with values of 818.07 and 146.08µg/g, respectively, Additionally, the ratio of EAA/total AAs determined in *S. aegyptiaca* and *S. monoica* were 16.7 and 17.5%, respectively, indicated that both species have resemblance with almost free AAs contents. Moreover, the current results showed that glutamic acid and aspartic acid were the major amino acids in all species and accounted for 50–63% and 10–22% of the total AA, respectively. 

With exception of the huge amounts of these amino acids and the absence of cystine in all investigated halophytes, other amino acids (non-EAA) were detected in considerable amounts ranging from 55.94 µg/g (arginine) to 12.93 µg/g (alanine). In contrast, histidine and threonine (EAA) were recorded at the lowest values (less than 0.3 and 0.8% of the total AAs, respectively), compared with other EAA in all species.

The AAs profiles in the present study of *S. aegyptiaca* and *S. monoica* were found to be quite different when compared to non-salt stressed related species [10,29]. El-Tantawy reported that aspartic acid, glutamic acid, proline, glycine, alanine, cysteine, and threonine were completely absent in *S. aegyptiaca* while Elsharabasy et al., identified methionine and histidine as the most abundant AAs with the absence of valine, isoleucine, and phenylalanine in *S. monoica*. The present study agreed with the previous findings that the change in AA constituents in response to salinity is considered an important factor since negatively charged ions (aspartate and glutamate) played a significant role in osmoregulation [30]. Earlier reports indicated that raising the salinity immediately increased the levels of aspartate, glutamate, glycine, histidine, lysine, and arginine. Moreover, proline is a well-known amino acid involved in plant stress tolerance as an osmolyte as well as antioxidant. Additionally, the synthesis of alanine reduces sodium to potassium ratios in plants, while the accumulation of isoleucine and phenylalanine promotes glycolysis to alleviate salt stress [31]. There is a possibility that glycine, an amino acid earlier reported to have unique osmotic properties [32], could help protect the cells of halophytes against osmotic damage. In general, AAs’ osmotic activity is attributable to their dipolar zwitterion properties, and their solubility in water.

### 2.4. Free Fatty Acid Compositions

The fatty acids (FAs) profiles of the halophytes are comprised of 7 to 14 FAs components ranging from C12 to C26 accounting for the total FAs ranging from 88.48 to 98.8% (Table 4). *S. monoica* and *Z. album* were recorded with the largest concentration of SFA (~51%) with respect to their total FAs. Conversely, *A. ehrenergii* and *S. aegyptiaca* were characterized by their high percentage of unsaturated FAs which represent 55.33 and 51.49% of the total FAs, respectively. For instance, the unsaturation of various halophytes has been reported in a range of 54–74% [33]. The most abundant SFA in all the investigated halophytes was palmitic acid (16:0) with a maximum value of 49.49% (*S. monoica*), which had been previously reported to be the most predominant fatty acid in halophytes [34]. Omega-9 oleic acid (C18:1) was the major MUFA (24.14–5.68%) and linoleic acid (C18:2) as omega-6 was the most predominant PUFA (28.63–15.54%), where both acids were prevalent in all investigated halophytes. Furthermore, *A. ehrenergii* and *S. monoica* possessed relatively high levels of the C18 conjugated FAs as trienoic (α-linolenic acid, C18:3, n-3) with proportions of 15.12 and 18.97%, respectively, and dienoic (linolenic acid, C18:2, n-6) with proportions of 28.63 and 22.49%, respectively. Besides both being essential omega-FAs, they have several described beneficial biological and pharmacological values including anti-inflammatory activity [35,36]. 

The highest omega-6 was recorded from *A. ehrenergii* (28.63%), followed by 24.14% omega-9 from *Z. album*, 18.97% omega-3 from *S. monoica*, and 2.5% omega-7 from *Z. album.* However, *S. aegyptiaca* has good proportions of omega-9 (22.81%) and omega-6 (21.03%), as well as has a significant nutrition ratio of omega-6/omega-3 (2.89:1) recommended in the diet. Many studies showed that a high omega-6/omega-3 ratio (~16:1), as found in today’s Western diets promotes the pathogenesis of many diseases including cardiovascular disease, cancer, and inflammatory and autoimmune diseases whereas a low omega-6/omega-3 ratio (~3:1 to 4:1) exerts suppressive effects [37].

Surprisingly in this study, the long-chain PUFA arachidonic acid (C20:4, n-6) and docosahexaenoic acid (C22:6, n-3) were only detected in *S. aegyptiaca* as traces. These fatty acids are generally major components of cell membranes and have particular importance to the brain and blood vessels that are essential for neurogenesis and brain development [38]. However, there was evidence that PUFA contributes greatly to the resistance of halophyte species to photo-inhibition, with their concentration increased in membrane lipids compared with non-halophytes, which enhances photosystem II’s tolerance to salt stress [39,40].

### 2.5. Hydrocarbons and Sterols Compositions

Terpenoids exhibited a great diversity in halophyte plants. In general terms, among the four investigated halophytes species, triterpenes (6 compounds) were the most unsaponifiable matter (USM) abundant, followed by sesquiterpenes (4 compounds), diterpenes (3 compounds), and one terpene lactone (Table 5). Diterpenes, neo-phytadiene (36.76%) and phytol (42.44%) with its degradation product phytone (8.31%), were the major constituents of *S. monoica*. Phytol which was already reported in other halophyte plants [41] is well known for its antioxidant activity as well as for its apoptotic effects in human gastric cancer cells [42]. Longipinane (15.75%) was the main sesquiterpene observed only in *Z. album* along with epizonarene (1.74%) and α-and β-eudesmol (4.42%). Several triterpenoids (β-amyrin, lupeol, botulin, uvaol, squalene, and 9,19-cycloart-23-ene-3,25-diol) were detected in all investigated halophytes with different amounts. The predominant triterpenes, lupeol (12.83%), and uvaol (11.94%) were recorded from *A. ehrenbergii* and *Z. album,* respectively. It can be clearly seen from the results that the content of terpenoids is the highest in *S. monoica* (100%) and the lowest in *S. aegyptiaca* (80.58%). Furthermore, a number of terpenoids (9 compounds) was identified in *Z. album*; contrastively, the least number (3 compounds) was identified in *A. ehrenbergii*. The latter halophyte was found to be rich in an interesting lipophilic constituent 9,19-cyclolanost-24-en-3β-ol (cycloartenol) with a value of 44.14%. This phytosterol was reported to have an antiproliferative effect on glioma U87 cells [43]. Other phytosterols such as stigmastanol, 24-ethylcholest-22-en-3-ol, stigmast-7-en-3-ol, and stigmast-3,5-diene were found only in *S. aegyptiaca* corresponding to 18.33,11.08, 5.10, and 2.15% of the total USM, respectively. Moreover, lanosterol (21.42%) and taraxasterol (5.12%) were found only in *Z. album*.

Recently, a number of studies have found a reduced risk of breast cancer and cardiovascular diseases with some phytosterols [44,45]. Other major components were identified such as 2,2,4-trimethyl-3-(3,8,12,16-tetramethyl-heptadeca-3,7,11,15-tetraenyl)-cyclohexanol with a proportion of 9.42% from *A. ehrenbergii,* and behenic alcohol (5.33%) and α-eudesmol (4.08%) from *Z. album*.

### 2.6. Antioxidant Activity

In addition to their morphological and physiological adaptations to high salinity, halophytes have developed different antioxidative stress defense mechanisms, including producing biologically active metabolites to resist and quench reactive oxygen species (ROS) toxicity [46]. In this study, the DPPH free radical scavenging assay was used to evaluate the antioxidant activity of the MeOH extract, USM, FAs, and phenolic contents (Table 6). The results interestingly demonstrated that *S. monoica* extract and its phenolic content were the most potent antioxidant with IC_50_ values of 9.0 and 8.0 μg/mL, compared to the antioxidant ascorbic acid (IC_50_ value of 10.6 μg/mL). In addition, the phenolic fraction of *S. aegyptiaca* exhibited high antioxidant activity with an IC_50_ value of 10.1 μg/mL. These antioxidant activities of *S. aegyptiaca* and *S. monoica* are attributed to the presence of high amounts of gallic acid and chlorogenic acid. The antioxidant effect of phenolic acids is thought to be based on their ability to directly scavenge free radicals, inhibit ROS-producing enzymes, and activate the antioxidant enzyme system to repair ROS-induced damage [47]. Conversely, the phenolic fractions of other halophytes are nearly inactive, while only *A. ehrenbergii* extract showed relatively weak activity with an IC_50_ value of 28.3 μg/mL. These findings match well with the previous studies [48,49].

### 2.7. Cytotoxic Activity

The MeOH extracts, USM, FAs, and phenolic contents were screened for their cytotoxicity against three human cancer cells: A549, Huh-7, and Caco2. The bioactive samples were assessed for their IC_50_ values (Table 6), where *S. aegyptiaca* crude extract showed a high cytotoxic effect against Huh-7 (11.3 μg/mL) and A-549 (14.5 μg/mL) cells, which may be induced by the USM content activity (IC_50_ 12.1 μg/mL). The same extract displayed a mild cytotoxic effect on Caco2 cells with an IC_50_ value of 24.8 μg/mL. However, the FAs and phenolic contents of *S. aegyptiaca* showed mild to weak activity on all tested cell lines with IC_50_ values ranging from 23.2 to 58.7 μg/mL. Unlike *S. aegyptiaca* extract, *S. monica* and *Z. album* extracts were resistant to all tested cell lines in this assay, and *A. ehrenbergii* showed the least cytotoxic extract on A-549 cells with an IC_50_ value of 50.0 μg/mL. Interestingly, the FA content of *Z. album* showed the highest cytotoxic activity against all cell lines with IC_50_ ranging from 7.4 to 11.8 μg/mL. In addition, significant cytotoxic effects were observed from the USM of *Z. album* against A-549 and Huh-7 cells with IC_50_ values of 13.2 and 11.3 μg/mL, respectively, and moderate cytotoxic potential against Caco2 cells with an IC_50_ value of 22.1 μg/mL. *S. monica* MeOH extract and chemical contents did not show anticancer properties with all tested cell lines except the FAs content which exhibited mild activity against liver Huh7 and lung A-549 cells with IC_50_ values of 27.9 and 30.3 μg/mL, respectively. These results are well agreed with those reported of Iraq *S. monica* extract against liver cancer cells [50].

## 3. Materials and Methods

### 3.1. Plant Materials

The leaves of four halophyte species, *A. ehrenbergii*, *S. aegyptiaca*, *S. monoica*, and *Z. album* were collected by hand from the Southern Corniche of Jeddah (21.254022 N; 39.134704 E), Saudi Arabia. The samples were identified by Marine Biology Department, Faculty of Marine Sciences, King Abdulaziz University, where voucher specimens of *Anabasis ehrenbergii* (Schweinf), *Suaeda aegyptiaca* (Hasselq. Zoh.), *Suaeda monoica* (Forsk), and *Zygophyllum album* (L.) have been deposited. All samples were rinsed with distilled water and dried in an oven at 60 °C. Dried powdered samples (1 g of each plant) were extracted with 20 mL of methanol/water mixture (70:30; *v*/*v*) at room temperature, for 24 h. The extracts were then filtered through a Whatman No 4 filter paper. The filtrates were concentrated using a vacuum rotary evaporator at 40 °C to yield crude extracts which were then stored at −27 °C until chemical analyses.

### 3.2. GC-MS Analysis of Monosaccharide Compositions

The halophytic samples (2.5 g) were extracted twice in 40 mL of boiling water, and the polysaccharides were precipitated by ethanol [51]. The polysaccharide precipitates were washed successively with acetone and ethanols then the precipitates were freeze-dried. The dry polysaccharide powder of each plant (1.0 mg) was heated in 0.5 mL oximination reagent (2.5% hydroxylamine hydrochloride in pyridine) at 80 °C for 1 h. After cooling, 1.0 mL of silylation reagent (trimethylchlorosilane: *N*,*O*-bis(trimethylsilyl)acetamide; 1:5, *v*:*v*) was added, and the mixture was kept at 80 °C for 30 min. The silylated components were analyzed by HP (6890 series) GC equipped with an FID detector under the following condition: column ZB-1701 (30 m × 0.25 mm, 0.5 μm, 14% cyanopropylphenylmethylpolysiloxane). Helium as a carrier gas was used at a flow rate of 1.2 mL/min. The temperature was set at 150–200 °C, at a rate of 7 °C min^−1^.

### 3.3. HPLC Analysis of Amino Acids Compositions

The total protein (%) of each halophyte species was determined using the methods described by AOAC (1990) [52]. The acid hydrolysis of the protein samples was carried out and the compositions of amino acids were determined using the Agilent method [53]. In this method, each protein sample was weighted into a hydrolyzed tube and heated with 6N HCl in a 110 °C oven for 24 h. After cooling, the contents were quantitatively dissolved in HPLC-grade water. Aliquots of hydrolysate (1 mL), together with appropriate standards were loaded into reaction vials. Before injection, amino acids were derivatized online using *o*-phthaldehyde (OPA). HPLC chromatographic analysis was carried out using an Agilent 1260 series using the Eclipse Plus C18 column (4.6 mm × 250 mm i.d., 5 μm). The mobile phase consisted of buffer (sodium phosphate dibasic and sodium borate), pH 8.2, and ACN:MeOH:H_2_O 45:45:10 at a flow rate of 1.5 mL/min. Fluorescence detection was carried out at 340 (excitation) and 450 nm (emission). The UV diode array detector was used to determine cystine at 338 nm. Before injecting the samples, the lysine standard was used for calibration.

### 3.4. HPLC Analysis of Phenolic Compounds

The quantitative estimation of phenolic acids: *p*-coumaric, caffeic, chlorogenic, syringic, ferulic, cinnamic, ellagic, gallic and methyl gallate, vanillin, pyrocatechol, and flavonoids (catechin, quercetin, rutin, naringenin, kaempferol, and hesperetin) in MeOH extracts of the halophyte plants was determined using reversed-phase HPLC. Agilent HPLC-1260 series with a C18 column (4.6 × 250 mm, 5 μm) was used with the mobile phase of water (A) and 0.02% trifluoroacetic acid in acetonitrile (B) at a flow rate of 1 mL/min. The mobile phase was programmed consecutively in a linear gradient as: 0–5 min (80% A); 5–8 min (40% A); 8–12 min (50% A); 12–14 min (80% A); and 14–16 min (80% A). The UV detector was monitored at λ 280 nm. Peaks were identified by congruent retention times and compared with those of the standards [54].

### 3.5. GC-MS Analysis of Lipoidal Compositions

#### 3.5.1. Extraction of Lipoidal Matters 

The powder of each halophyte sample (100 g) was extracted with a mixture of MeOH/CHCl_3_ (1:1, *v*:*v*) by soaking at room temperature. Extraction for each sample was repeated many times until exhausted. The combined solvent extracts for each plant sample were concentrated under reduced pressure at 40 °C until dryness. The resulted crude extracts were dissolved in water and fractionated with chloroform. The CHCl_3_ fractions were concentrated until dryness. 

#### 3.5.2. Unsaponifiable Matter (USM)

One gram of each CHCl_3_ fraction was saponified with alcoholic KOH (30 g KOH in 1000 mL EtOH) at 80 °C for 6 h under reflux. The USM matter was extracted with diethyl ether, washed several times with distilled water, and dried over anhydrous Na_2_SO_4_. Then the solvent was evaporated, and the USM content was subjected to the GC-MS analysis [55].

#### 3.5.3. Saponifiable Matter: Free Fatty Acids (FAs)

The alkaline aqueous solutions remaining after extraction of the USM were acidified with HCl to liberate the FAs which were then extracted several times with diethyl ether. The combined extracts were washed several times with distilled water and then filtered over anhydrous Na_2_SO_4_. The filtrates were evaporated to dryness as FAs (saponified) contents [56]. FAs were methylated by refluxing the saponified contents (each in 100 mL absolute MeOH with 3 mL of H_2_SO_4_) for 3 h. Then the contents were cooled and diluted with distilled water (about 100 mL) for diethyl ether extraction. The resultant extracts of saponifiable FAs-methyl ester (FAs-ME) were dried over anhydrous Na_2_SO_4_. The solvents were evaporated and the residues of FAs-ME were subjected to GC-MS analysis.

#### 3.5.4. GC-MS Analysis 

GC-MS analysis of the USM and FAs-ME fractions was carried out on a Hewlett-Packard 6890 GC equipped with an HP-5MS capillary column (30 m × 0.32 mm × 0.2 µm film thickness) and MS spectrometric detector. Helium was used as a carrier gas at a column head pressure of 60 kPa. The column oven temperature cycle was 50 °C for 10 min, then 50 to 310 °C gradually 3 °C/min, then 310 °C for 20 min. The identification of the constituents was performed by comparing their spectral fragmentation patterns with those of the available database libraries Wiley USA (Wiley Int.), and/or published data. Quantitative determination was carried out based on peak area integration [57].

### 3.6. Antioxidant Activity: Free Radical Scavenging Assay

The antioxidant activity of the MeOH extract and phenolic and lipoidal contents were evaluated by DPPH free radical scavenging method [58]. The DPPH free radical has a deep purple color and a characteristic absorbance peak at 517 nm. Briefly, the methanolic stock solution of 0.1 mM DPPH reagent was freshly prepared and 0.1 mL of sample and 0.9 mL methanol were added to 2 mL of 0.06 mM DPPH methanolic solution. After vortex-mixed for 10 s, the absorbance at 517 nm was determined with a spectrophotometer (UV-*vis* spectrophotometer). The addition of samples resulted in a decrease in the absorbance due to the scavenging activity of the oxidisable groups of a sample. All samples and the antioxidant control (ascorbic acid) were made in triplicate. The percentage of inhibition achieved by different concentrations of samples was calculated by the following equation: I (%) = (A_0_ − A)/A_0_ × 100, where (A_0_) is the absorbance of the control reaction and (A) is the absorbance of the examined samples. The values were calculated by regression analysis of the data for a series of diluted sample solutions.

### 3.7. Cytotoxicity Assay

Human lung cancer (A549), hepatocellular cancer (Huh-7 cells), and intestinal cancer (Caco2) cell lines were obtained from VACSERA Tissue Culture Unit (Cairo, Egypt). Cell culture media RPMI1640 and IMDM, HEPES buffer, and stable glutamine and penicillin/streptomycin were obtained from Lonza (Pharma & Biotech, Portsmouth, NH, USA). All cells were maintained at 37 °C in a humidified atmosphere with 5% CO_2_. Cytotoxic activity of the plant metabolites (MeOH extract and contents of phenolic, FAs, and USM) was evaluated using cell viability assay [59], and vinblastine sulfate was used as a positive control. In brief, after the end of the incubation period, media were aspirated and the crystal violet solution (1%) was added to each well for at least 30 min. The stain was removed and the plates were rinsed using tap water until all excess stain is removed. Glacial acetic acid (30%) was then added to all wells and mixed thoroughly, and then the absorbance was measured at λ490 nm using a microplate reader (Tecan, Inc., Morrisville, NC, USA). The relation between surviving cells and sample concentration is plotted to get the survival curve of each tumor cell line after treatment with the plant metabolite samples. The 50% inhibitory concentration (IC_50_) was estimated from graphic plots of the dose–response curve for each concentration using Graphpad Prism software (San Diego, CA, USA). The data presented are the mean of at least three separate experiments. 

## 4. Conclusions

The Red Sea coastal area exhibits unique biodiversity with mangroves and halophytes in particular. Over the centuries, many of these species have been used for therapeutic purposes, and more recently, herbal medicines and food supplements have become more popular worldwide. The established procedure herein allowed investigation of the chemical composition of four understudied halophyte plants; *A. ehrenbergii*, *S. aegyptiaca*, *S. monoica*, and *Z. album*. Knowledge of both the phytochemical constituents and their amounts in these plant species might expand the basis for their future phytochemical exploration and food applications. Additionally, some of the crude extracts and fractions of these halophyte plants have significant antioxidant and cytotoxic properties; therefore, coupling the chemical composition of these plants to their biological activities could support a more effective isolation process that focuses on their predicted bioactive principles, ultimately improving drug discovery.

## Figures and Tables

**Table 1:** Monosaccharide compositions of polysaccharides from the Red Sea halophytes *.

Sugar	*Chenopodiaceae*			*Zygophyllaceae*
	*A. ehrenbergii*	*S. aegyptiaca*	*S. monoica*	*Z. album*
	Relative (%)			
Arabinose	-	-	-	36.67
Xylose	0.95	-	10.7	0.68
Ribose	0.65	8.79	26.0	-
Mannose	2.25	44.15	-	2.60
Rhamnose	3.00	3.35	0.68	-
Galactose	54.74	9.63	4.96	8.53
Glucose	14.79	16.3	33.0	31.52
Fucose	-	-	-	0.24
Glucouronic acid	-	0.19	0.62	3.09
Galacturonic acid	1.90	0.16	-	0.48

* Means values (*n* = 2).

**Table 2:** Phenolic contents of the investigated Red Sea halophytes (mg/g; *n* = 2).

Compound	*A. ehrenbergii*	*S. aegyptiaca*	*S. monoica*	*Z. album*
Cinnamic acid	0.003	0.02	0.02	0.004
Coumaric acid	0.38	0.79	2.80	0.37
Coffeic acid	1.1	0.13	-	-
Ferulic acid	0.13	2.15	-	0.40
Syringic acid	2.4	2.47	1.07	0.39
Chlorogenic acid	5.07	-	7.20	-
Ellagic acid	9.96	-	1.12	0.61
Gallic acid	4.95	41.72	47.48	0.57
Methyl gallate	1.74	0.04	0.16	-
Vanillin	0.38	0.77	0.18	0.16
Pyrocatechol	1.26	-	1.68	-
Catechin	0.21	0.04	0.63	-
Naringenin	11.88	1.50	1.49	6.42
Quercetin	0.38	0.09	0.02	0.06
Kaempferol	0.1	-	0.13	0.32
Rutin	4.79	0.1	0.59	5.17
Hesperetin	-	-	0.1	-

**Table 3:** Protein content (%) and amino acid compositions (µg/g DW) of the Red Sea halophytes.

Amino acids (AAs) *	*A. ehrenbergii*	*S. aegyptiaca*	*S. monoica*	*Z. album*
Total protein	6.33	7.6	8.28	5.72
Essential amino acids (EAA)				
Histidine	2.59	3.77	2.56	1.05
Leucine	47.92	44.75	51.95	32.61
Iso-leucine	15.75	15.08	15.18	20.69
Lysine	24.96	33.83	34.66	19.82
Methionine	39.15	28.25	30.65	29.62
Phenylalanine	77.13	55.29	59.08	64.41
Valine	17.02	14.25	14.73	17.65
Threonine	6.16	9.58	6.09	4.71
Non-essential amino acids (Non-EAA)				
Alanine	17.7	20.04	14.08	12.93
Arginine	28.65	16.32	16.62	55.94
Aspartic acid	94.81	180.64	274.59	146.08
Glutamic acid	448.13	702.98	612.18	818.07
Glycine	25.88	22.34	23.14	17.41
Proline	23.35	39.66	28.74	21.56
Serine	17.75	16.95	28.3	16.42
Tyrosine	14.11	20.72	16.73	19.93
∑AAs	901.06	1224.45	1229.28	1298.9
∑EAA	230.68	204.8	214.9	190.56
∑Non-EAA	670.38	1019.65	1014.38	1108.34
Ratio EAA/non-EAA	0.344	0.201	0.219	0.172
EAA/total AAs (%)	25.5	16.7	17.5	14.7

* Tryptophan has not been measured; means (*n* = 2).

**Table 4:** Fatty acid (FA) compositions (relative %; *n* = 2) of the investigated Red Sea halophytes.

Identified FAs	*A. ehrenbergii*	*S. aegyptiaca*	*S. monoica*	*Z. album*	Identified FAs	*A. ehrenbergii*	*S. aegyptiaca*	*S. monoica*	*Z. album*
*1. Saturated FAs (SFA)*					*2. Unsaturated FAs (UFA)*				
Lauric acid (C12:0)	1.3	-	-	0.65	5-Dodecenoic acid (12:1, n-7)	1.3	-	-	0.65
Myristic acid (C14:0)	5.05	2.79	0.85	3.53	Palmitoleic acid (C16:1, n-7)	-	-	-	1.85
Pentadecanoic acid (C15:0)	-	1.07	0.54	0.93	10*Z*-Heptadecenoic acid (C17:1, n-7)	-	0.38	-	-
Palmitic acid (C16:0)	23.94	26.34	49.49	33.16	α-Linolenic acid (C18:3, n-3)	15.12	6.9	18.97	-
14-Methylpalmitic acid (17:0)	2.2	-	-	-	Linoleic acid (C18:2, n-6)	28.63	20.83	22.49	15.54
Margaric acid (C17:0)	-	0.77	-	0.74	Oleic acid (C18:1, n-9)	10.28	22.05	5.68	24.14
Stearic acid (C18:0)	4.52	2.19	0.78	5.01	Arachidonic acid (20:4, n-6)	-	0.2		-
Arachidic acid (C20:0)	2.33	0.77		1.8	11*Z*-Eicosenoic acid (C20:1, n-9)	-	0.76		-
Behenic acid (C22:0)	2.39	2.46		4.11	Docosahexaenoic acid (DHA) (22:6, n-3)	-	0.37		-
Lignoceric acid (C24:0)	-	0.6		1.08	∑ω9	10.28	22.81	5.68	24.14
Cerotic acid (26:0)	-	-		0.41	∑ω7	1.30	0.38	-	2.50
*∑*SFA	41.73	36.99	51.66	51.42	∑ω6	28.63	21.03	22.49	15.54
					∑ω3	15.12	7.27	18.97	-
					ω6:ω3	1.89	2.89	1.19	-
					*∑*UFA	55.33	51.49	47.14	42.18
					*∑*PUFA	43.48	29.06	41.46	15.54
					*∑*FAs	97.06	88.48	98.8	93.6

**Table 5:** Unsaponifiable matters (USM, relative %; *n* = 2) of the investigated Red Sea halophytes.

Compound	*A. ehrenbergii*	*S. aegyptiaca*	*S. monoica*	*Z. album*	Compound	*A. ehrenbergii*	*S. aegyptiaca*	*S. monoica*	*Z. album*
*1. Hydrocarbon compounds*					Uvaol				11.94
5-Eicosene	-	3.16	-	-	9,19-Cycloart-23-ene-3,25-diol				2.03
Pentacosane	-	0.72	-	-	9,19-Cyclolanost-24-en-3β-ol	44.11	4.13		0.46
Hexacosane	-	0.87	-	-	24-Methyl-9,19-cyclolanost-25-en-3β–ol	3.5	5.64		
Heptacosane	-	2.73	-	0.49	24-Methyl-9,19-cyclolanost-24-en-3β–ol				0.61
Nonacosane	-	1.24	-	-	Cholesterol	0.96			
Pentacosane	-	0.72	-	-	Campesterol	1.22			3.98
γ-Asarone				2.49	Stigmasterol	5.43	8.98	1.11	2.24
*2. Oxygenated compounds*					β-Sitosterol			0.41	
Neo-phytadiene	-	1.12	36.76	-	γ–Sitosterol	14.15	16.65	-	10.6
Phytone		1.12	8.31	0.28	Stigmastanol		18.33		
Phytol	3.11	2.99	42.44	1.65	Stigmast-7-en-3β-ol		5.10		
Behenic alcohol	-	-	-	5.33	Stigmast-3,5-diene	*-*	2.15	*-*	*-*
α-Eudesmol	-	-	-	4.08	Taraxasterol				5.12
β-Eudesmol	-	-	-	0.34	Lanosterol				21.42
2,2,4-Trimethyl-3-(3,8,12,16-tetramethyl-heptadeca-3,7,11,15-tetraenyl)-cyclohexanol	9.42	-	-	-	24-Ethylcholest-22-en-3α-ol		11.08		
Dihydroactinidiolide			3.67	0.41	∑Sterols and terpenes	94.63	80.58	100	87.57
Epizonarene	-	-	-	1.74	∑USM compounds	94.63	90.02	100	90.55
Longipinane				15.75					
β-Amyrin	-	-	3.09	-					
Squalene	0.93	-	-	-					
Lupeol	12.83	3.29	4.21	-					

**Table 6:** Antioxidant and cytotoxic activities (IC_50_ µg/mL, *n* = 3) of the halophytic MeOH extracts and lipoidal and phenolic contents.

Sample	Antioxidant	Cytotoxic Activity		
		A-549	Huh-7	CACO-2
***A. ehrenbergii***				
MeOH extract	28.3 ± 2.1	50.0 ± 1.9	-	-
FAs content	-	29.3 ± 1.7	23.6 ± 0.9	42.7 ± 2.1
USM content	-	-	-	-
Phenolic content	72.4 ± 1.9	-	-	-
***S. aegyptiaca***				
MeOH extract	74.0 ± 4.7	14.5 ± 0.6	11.3 ± 0.7	24.8 ± 2.6
FAs content	-	33.5 ± 1.5	47.8 ± 2.1	58.7 ± 2.4
USM content	-	12.1 ± 1.0	12.1 ± 0.7	23.2 ± 1.5
Phenolic content	10.1 ± 0.6	41.2 ± 1.9	28.9 ± 1.6	46.4 ± 3.9
***S. monoica***				
MeOH extract	9.0 ± 0.5	-	-	-
FAs content	-	30.3 ± 1.0	27.9 ± 1.2	50.8 ± 2.4
USM content	-	-	-	-
Phenolic content	8.0 ± 0.3	54.7 ± 1.8	-	-
***Z. album***				
MeOH extract	-	-	-	-
FAs content	-	10.8 ± 0.5	7.4 ± 0.3	11.8 ± 0.7
USM content	-	13.2 ± 0.8	11.3 ± 0.5	22.1 ± 1.1
Phenolic content	78.4 ± 2.8	-	-	-
**Ascorbic acid** (control)	10.6 ± 0.8			

## Data Availability

Data are available from authors on request.

## Supplementary Materials

The following are available online at https://www.mdpi.com/article/10.3390/molecules27113415/s1, Figures S1–S4: Monosaccharide compositions spectra; Figures S5–S8: Amino acids compositions spectra; Figures S9–S12: phenolic compositions spectra; Figures S13–S16: Fatty acids compositions spectra; Figures S17–S20: Unsaponifiable matter compositions spectra.

## References

[1] Flowers T.J., Galal H.K., Bromham L. (2010). Evolution of halophytes: Multiple origins of salt tolerance in land plants. Funct. Plant Biol..

[2] Hasegawa P.M., Bressan R.A., Zhu J.K., Bohnert H.J. (2000). Plant cellular and molecular responses to high salinity. Ann. Rev. Plant Biol..

[3] James R.A., Rivelli A.R., Munns R., von Caemmerer S. (2002). Factors affecting CO_2_ assimilation, leaf injury and growth in salt-stressed durum wheat. Func. Plant Biol..

[4] Ksouri R., Ksouri W.M., Jallali I., Debez A., Magné C., Hiroko I., Abdelly C. (2012). Medicinal halophytes: Potent source of health promoting biomolecules with medical, nutraceutical and food applications. Crit. Rev. Biotechnol..

[5] Anjum N.A., Ahmad I., Valega M., Mohmood I., Gill S.S., Tuteja N., Duarte A.C., Pereira E. (2014). Salt marsh halophyte services to metal-metalloid remediation: Assessment of the processes and underlying mechanisms. Crit. Rev. Environ. Sci. Technol..

[6] Bandaranayake W.M. (1998). Traditional and medicinal uses of mangroves. Mangroves Salt Marshes.

[7] Chandrasekaran M., Kannathasan K., Venkatesalu V. (2008). Antimicrobial activity of fatty acid methyl esters of some members of Chenopodiaceae. Z. Naturforsch. C.

[8] Al-Said M.S., Siddiqui N.A., Mukhair M.A., Parvez M.K., Alam P., Ali M., Haque A. (2017). A novel monocyclic triterpenoid and a norsesquaterpenol from the aerial parts of *Suaeda monoica* Forssk. ex JF Gmel with cell proliferative potential. Saudi Pharm. J..

[9] Parvez M.K., Al-Dosari M.S., Rehman M.T., Alajmi M.F., Alqahtani A.S., AlSaid M.S. (2021). New terpenic and phenolic compounds from *Suaeda monoica* reverse oxidative and apoptotic damages in human endothelial cells. Saudi Pharm. J..

[10] Elsharabasy F.S., Metwally N.S., Mahmoud A.H., Soliman M.S., Youness E.R., Farrag A.H. (2019). Phytoconstituents and hepatoprotective effect of *Suaeda monoica* Forssk and *Suaeda pruinosa* Lange. Biomed. Pharmacol. J..

[11] Ghazanfar S.A. (1994). Handbook of Arabia Medicinal Plant.

[12] Albosof F., Hoseini S.A., Siahpoush A., Malayeri A.R., Haghighizadeh M.H. (2018). Anti-diabetic effects of *S. aegyptiaca* extract on streptozotocin-nicotinamide induced type 2 diabetes rats. J.Contemp. Med. Sci..

[13] Mohammadi M.S., Bush S., Hosseini R.S., Karimi S., Mohammadipour N. (2016). Total phenolic and flavonoid contents and antioxidant activity of four medicinal plants from Hormozgan province, Iran. Res. J. Pharmcogn..

[14] Bellakhdar J., Claisse R., Fleurentin J., Younos C. (1991). Repertory of standard herbal drugs in the Moroccan pharmacopoea. J. Ethnopharmacol..

[15] Degen A.A., Kam M., Khokhlova I.S., Zeevi Y. (2000). Fiber digestion and energy utilization of fat sand rats (*Psammomys obesus*) consuming the chenopod *Anabasis Articulata*. Physiol. Biochem. Zool..

[16] Senhaji S., Lamchouri F., Toufik H. (2020). Phytochemical content, antibacterial and antioxidant potential of endemic plant anabasis aretioïdes coss. & moq.(Chenopodiaceae). BioMed Res. Int..

[17] Hammiche V., Maiza K. (2006). Traditional medicine in Central Sahara: Pharmacopoeia of Tassili N’ajjer. J. Ethnopharmacol..

[18] Moustafa A.M.Y., Khodhair A.I., Hammouda F.M., Husseiny H.A. (2007). Phytochemical and toxicological studies of *Zygophyllum album* L.F. J. Pharmacol. Toxicol..

[19] El-Ghoul J., Boughattas N.A., Ben-Attia M. (2012). Antihyperglycemic and antihyperlipidemic activities of ethanolic extract of *Zygophyllum album* in streptozotocin-induced diabetic mice. Toxicol. Ind. Health.

[20] El-Ghoul J., Smiri M., Ghrab S., Boughattas N.A., Ben-Attia M. (2012). Antihyperglycemic, antihyperlipidemic and antioxidant activities of traditional aqueous extract of *Zygophyllum album* in streptozotocin diabetic mice. Pathophysiology.

[21] Elgamal M.H.A., Shaker K.H., Pöllmann K., Seifert K. (1995). Triterpenoid saponins from *Zygophyllum* species. Phytochemistry.

[22] Haseanean H.A., El-Hamouly M.M.A., El-Moghazy S.A., Bishay D.W. (1993). 14-Decarboxyquinovic and quinovic acid glycosides from *Zygophyllum album*. Phytochemistry.

[23] Fu J., Shao J., Wang M., Zhang G., Fang Y. (2020). Optimization of extraction of polysaccharides from Suaeda salsa (L.) Pall. by ultrasonic: Characterization, purification and antioxidant assessment. E3S Web. Conf..

[24] Mzoughi Z., Abdelhamid A., Rihouey C., Le Cerf D., Bouraoui A., Majdoub H. (2018). Optimized extraction of pectin-like polysaccharide from *Suaeda fruticosa* leaves: Characterization, antioxidant, anti-inflammatory and analgesic activities. Carbohydr. Polym..

[25] Flowers T.J., Colmer T.D. (2015). Plant salt tolerance: Adaptations in halophytes. Ann. Bot..

[26] Qasim M., Abideen Z., Adnan M.Y., Gulzar S., Gul B., Rasheed M., Khan M.A. (2017). Antioxidant properties, phenolic composition, bioactive compounds and nutritive value of medicinal halophytes commonly used as herbal teas. S. Afr. J. Bot..

[27] Lopes M., Sanches-Silva A., Castilho M., Cavaleiro C., Ramos F. (2021). Halophytes as source of bioactive phenolic compounds and their potential applications. Crit. Rev. Food Sci. Nutr..

[28] Ahmed F.A., Lotfy R.A. (2015). Phytochemical evaluation of some selected medicinal plants growing wildly in Southeastern Egypt. Middle East J. Appl. Sci..

[29] El-Tantawy H. (2002). The nutritional value of some desert plants in Kuwait, Arabian Pennisula. Taeckholmia.

[30] Rani G. (2007). Changes in protein profile and amino acids in *Cladophora vagabunda* (Chlorophyceae) in response to salinity stress. J. Appl. Psychol..

[31] Wu D., Cai S., Chen M., Ye L., Chen Z., Zhang H., Dai F., Wu F., Zhang G. (2013). Tissue metabolic responses to salt stress in wild and cultivated barley. PLoS ONE.

[32] Eklund M., Bauer E., Wamatu J., Mosenthin R. (2005). Potential nutritional and physiological functions of betaine in livestock. Nutr. Res. Rev..

[33] Weber J., Ansari R., Gul B., Khan M.A. (2007). Potential of halophytes as source of edible oil. J. Arid Environ..

[34] Hamed K.B., Youssef N.B., Ranieri A., Zarrouk M., Abdelly C. (2005). Changes in content and fatty acid profiles of total lipids and sulfolipids in the halophyte *Crithmum maritimum* under salt stress. J. Plant Physiol..

[35] Erdinest N., Shmueli O., Grossman Y., Ovadia H., Solomon A. (2012). Anti-inflammatory effects of alpha linolenic acid on human corneal epithelial cells. Investig. Ophthalmol. Vis. Sci..

[36] Nagao K., Yanagita T. (2005). Conjugated fatty acids in food and their health benefits *J*. Biosci. Bioeng..

[37] Simopoulos A.P. (2002). The importance of the ratio of omega-6/omega-3 essential fatty acids. Biomed. Pharmacother..

[38] Crawford M.A. (2000). Placental delivery of arachidonic and docosahexaenoic acids: Implications for the lipid nutrition of preterm infants. Am. J. Clin. Nutr..

[39] Sui N., Li M., Li K., Song J., Wang B.S. (2010). Increase in unsaturated fatty acids in membrane lipids of *Suaeda salsa* L. enhances protection of photosystem II under high salinity. Photosynthetica.

[40] Sui N., Han G. (2014). Increases of unsaturated fatty acids in membrane lipids protects photosystem II from photoinhibition under salinity in different halophytes. J. Agric. Sci..

[41] Faustino M.V., Faustino M.A., Pinto D.C. (2019). Halophytic grasses, a new source of nutraceuticals? A review on their secondary metabolites and biological activities. Int. J. Mol. Sci..

[42] Olofsson P., Hultqvist M., Hellgren L.I., Holmdahl R., Jacob C., Kirsch G., Slusarenko A.J., Winyard P.G., Burkholz T. (2014). Phytol: A chlorophyll component with anti-inflammatory and metabolic properties. Recent Advances in Redox Active Plant and Microbial Products.

[43] Niu H., Li X., Yang A., Jin Z., Wang X., Wang Q., Yu C., Wei Z., Dou C. (2018). Cycloartenol exerts anti-proliferative effects on Glioma U87 cells via induction of cell cycle arrest and p38 MAPK-mediated apoptosis. J. BUON.

[44] Bruce J., Grattan J. (2013). Plant sterols as anticancer nutrients: Evidence for their role in breast cancer. Nutrients.

[45] Woyengo T.A., Ramprasath V.R., Jones P.J. (2009). Anticancer effects of phytosterols. Eur. J. Clin. Nutr..

[46] Petropoulos S.A., Karkanis A., Martins N., Ferreira I. (2018). Halophytic herbs of the Mediterranean basin: An alternative approach to health. Food Chem. Toxicol..

[47] Kilic K., Sakat M.S., Akdemir F.N.E., Yildirim S., Saglam Y.S., Askin S. (2019). Protective effect of gallic acid against cisplatin-induced ototoxicity in rats. Braz. J. Otorhinolar..

[48] Daffodil E.D., Rajalakshmi K., Mohan V.R. (2012). Estimates total phenolic, flavonoid content and in vitro antioxidant activity of root of *Suaeda monaica* Forssk ex Gmel (Chenopodiaceae). Elixir. Appl. Bot..

[49] Roy M., Dutta T.K. (2021). Evaluation of Phytochemicals and Bioactive Properties in Mangrove Associate *Suaeda monoica* Forssk. ex JF Gmel. of Indian Sundarbans. Front. Pharmacol..

[50] Al-Shawi A.A., Hameed M.F., Ali N.H., Hussein K.A. (2020). Investigations of phytoconstituents, antioxidant and anti-liver cancer activities of *Saueda monoica* Forssk extracted by microwave-assisted extraction. Asian Pac. J. Cancer Prev..

[51] Kirk R., Sawer R. (1991). Pearson’s *Composition*and analysis of foods. Longman Scientific and Technical.

[52] AOAC (1990). Official Methods of Analysis.

[53] Henderson J.W., Ricker R.D., Bidlingmeyer B.A., Woodward C. (2000). Rapid, accurate, sensitive, and reproducible HPLC analysis of amino acids. Amino Acid Anal. Using Zorbax Eclipse-AAA Columns Agil..

[54] Kim K., Tsao R., Yang R., Cui S. (2006). Phenolic acid profiles and antioxidant activities wheat bran extract and the effect of hydrolysis conditions. Food Chem..

[55] Tsuda K., Sakai K., Tanabe K., Kishida Y. (1960). Steroid Studies. XVI.1 Isolation of 22-dehydrocholestrol from *Hypnea japonica*. J. Am. Chem. Soc..

[56] Farag R.S., Hallabo S.S., Hewedi F.M., Basyony A.E. (1986). Chemical Evaluation of Rapeseed. Fette Seifen Anstrichm..

[57] Adams R.P. (1989). Identification of Essential Oils by Ion Trap Mass Spectroscopy.

[58] El-Kassem L.T., Mohammed R.S., El-Souda S.S., El-Anssary A.A., Hawas U.W., Mohmoud K., Farrag A. (2012). Digalacturonide flavones from Egyptian *Lantana camara* flowers with in vitro antioxidant and in vivo hepatoprotective activities. Z. Naturforsch. C.

[59] Mosmann T. (1983). Rapid colorimetric assay for cellular growth and survival:application to proliferation and cytotoxicity assays. J. Immunol. Methods.

