# Supplementary material for: Phytochemical Compositions of Some Red Sea Halophyte Plants with Antioxidant and Anticancer Potentials
Source: Molecules. 2022 May 25;27(11):3415. doi: 10.3390/molecules27113415 (PMC9182077; doi:10.3390/molecules27113415)
Supplement: Supplementary file 1 [file molecules-27-03415-s001.zip › molecules-1682563-supplementary.pdf]

Supplementary Materials

Figure S1: Monosaccharide compositions spectrum of *Anabasis ehrenbergii*

Figure S2: Monosaccharide compositions spectrum of *Suaeda aegyptiaca*

Figure S3: Monosaccharide compositions spectrum of *Suaeda monoica*

Figure S4: Monosaccharide compositions spectrum of *Zygophyllum album*

Figure S5: Amino acids compositions spectrum of *Anabasis ehrenbergii*

Figure S6: Amino acids compositions spectrum of *Suaeda aegyptiaca*

Figure S7: Amino acids compositions spectrum of *Saueda monoica*

Figure S8: Amino acids compositions spectrum of *Zygophyllum album*

Figure S9: Phenolic compositions spectrum of *Anabasis ehrenbergii*

Figure S10: Phenolic compositions spectrum of *Suaeda aegyptiaca*

Figure S11: Phenolic compositions spectrum of *Saueda monoica*

Figure S12: Phenolic compositions spectrum of *Zygophyllum album*

Figure S13: Fatty acids compositions spectrum of *Anabasis ehrenbergii*

Figure S14: Fatty acids compositions spectrum of *Suaeda aegyptiaca*

Figure S15: Fatty acids compositions spectrum of *Saueda monoica*

Figure S16: Fatty acids compositions spectrum of *Zygophyllum album*

Figure S17: Unsaponifiable matter compositions spectrum of *Anabasis ehrenbergii*

Figure S18: Unsaponifiable matter compositions spectrum of *Suaeda aegyptiaca*

Figure S19: Unsaponifiable matter compositions spectrum of *Suaeda monoica*

Figure S20: Unsaponifiable matter compositions spectrum of *Zygophyllum album*

1. Polysaccharide analysis:

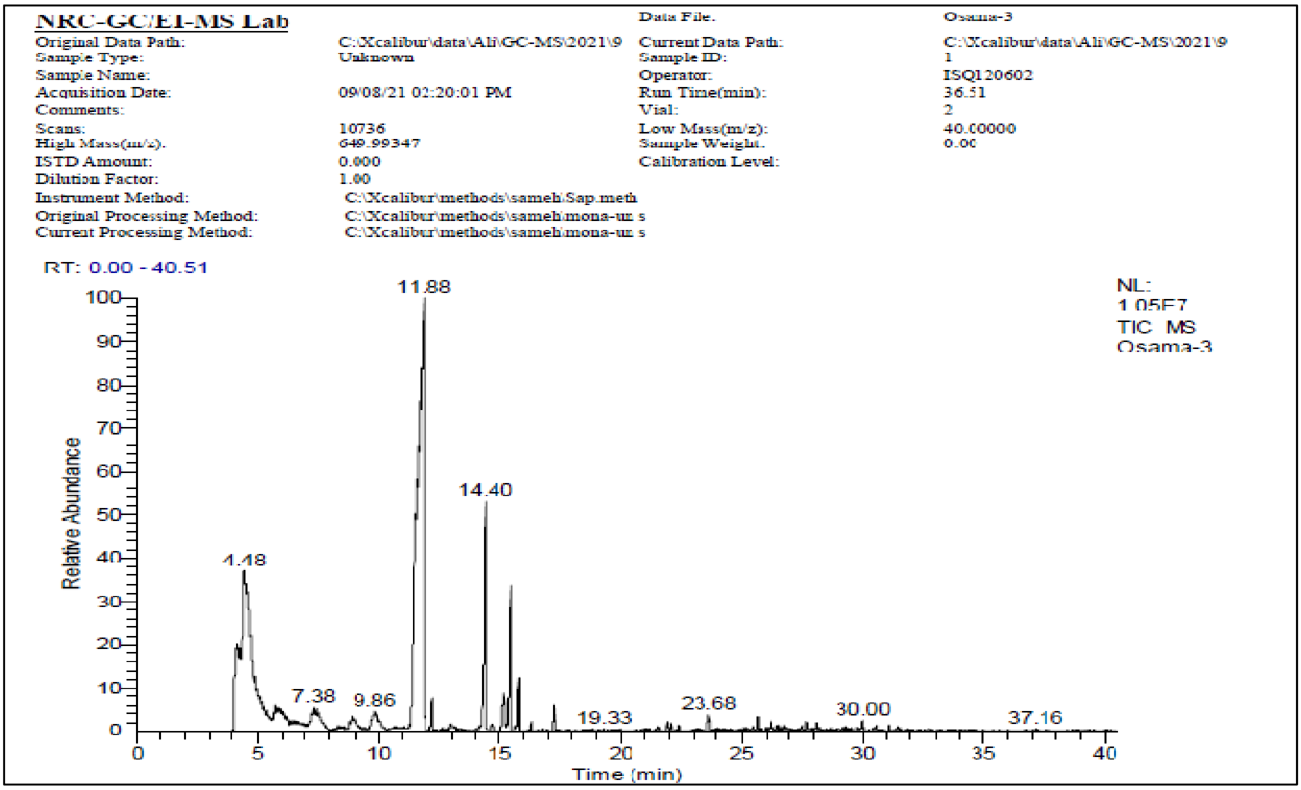

Figure S1: Monosaccharide compositions spectrum of *Anabasis ehrenbergii*

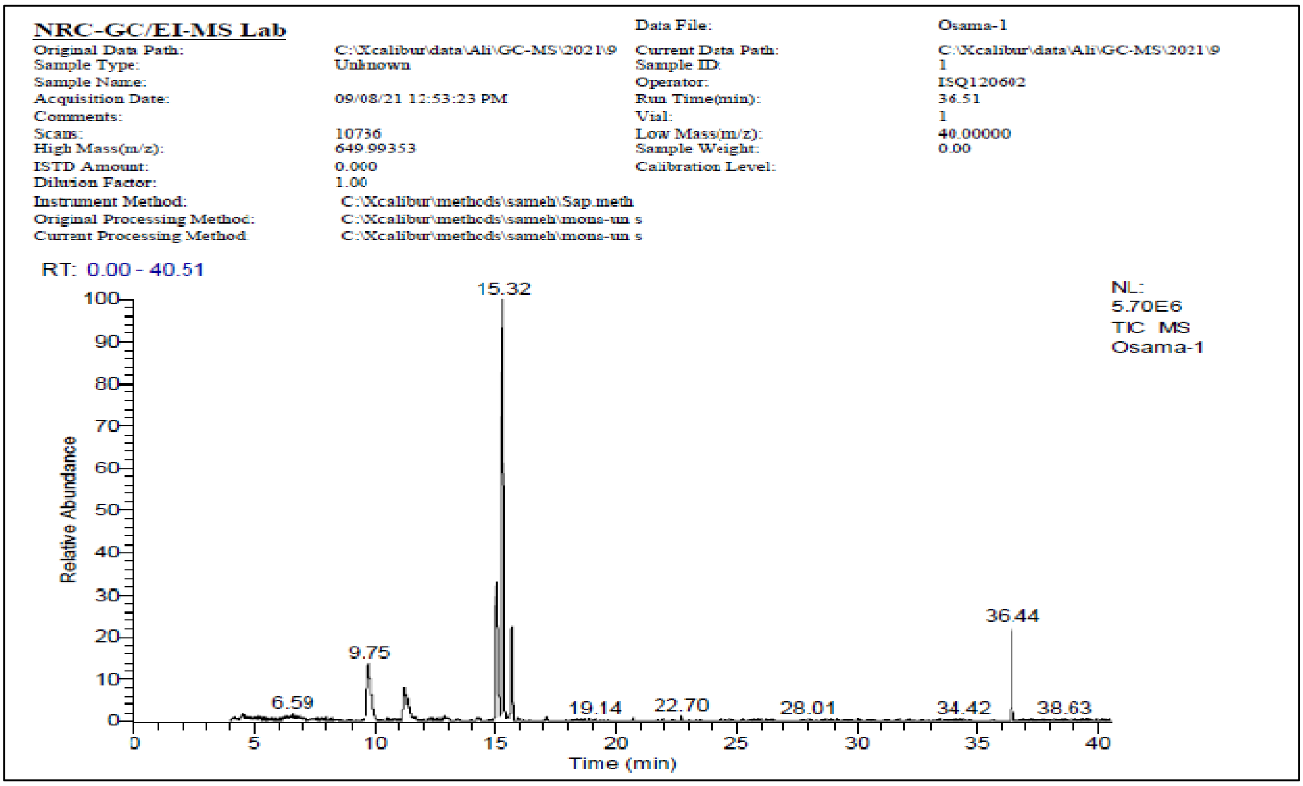

Figure S2: Monosaccharide compositions spectrum of *Suaeda aegyptiaca*

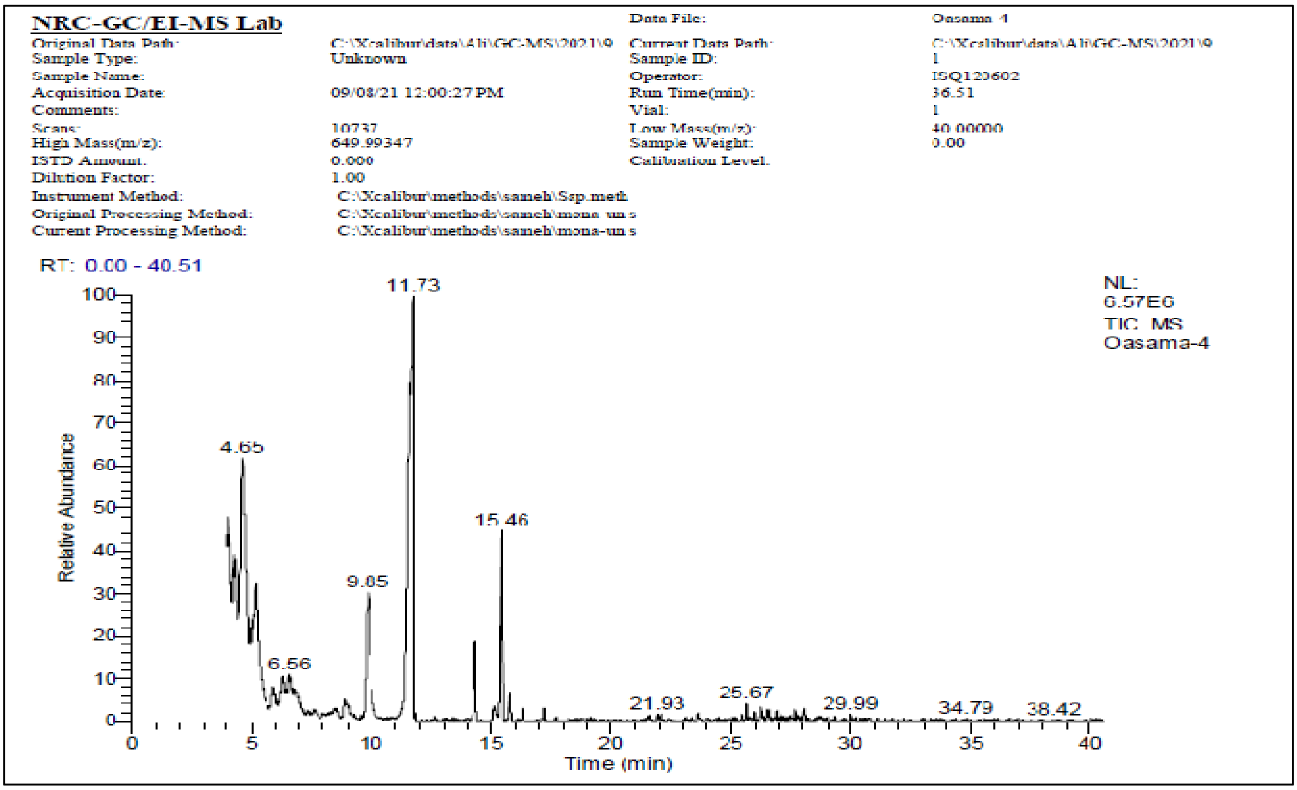

Figure S3: Monosaccharide compositions spectrum of *Suaeda monoica*

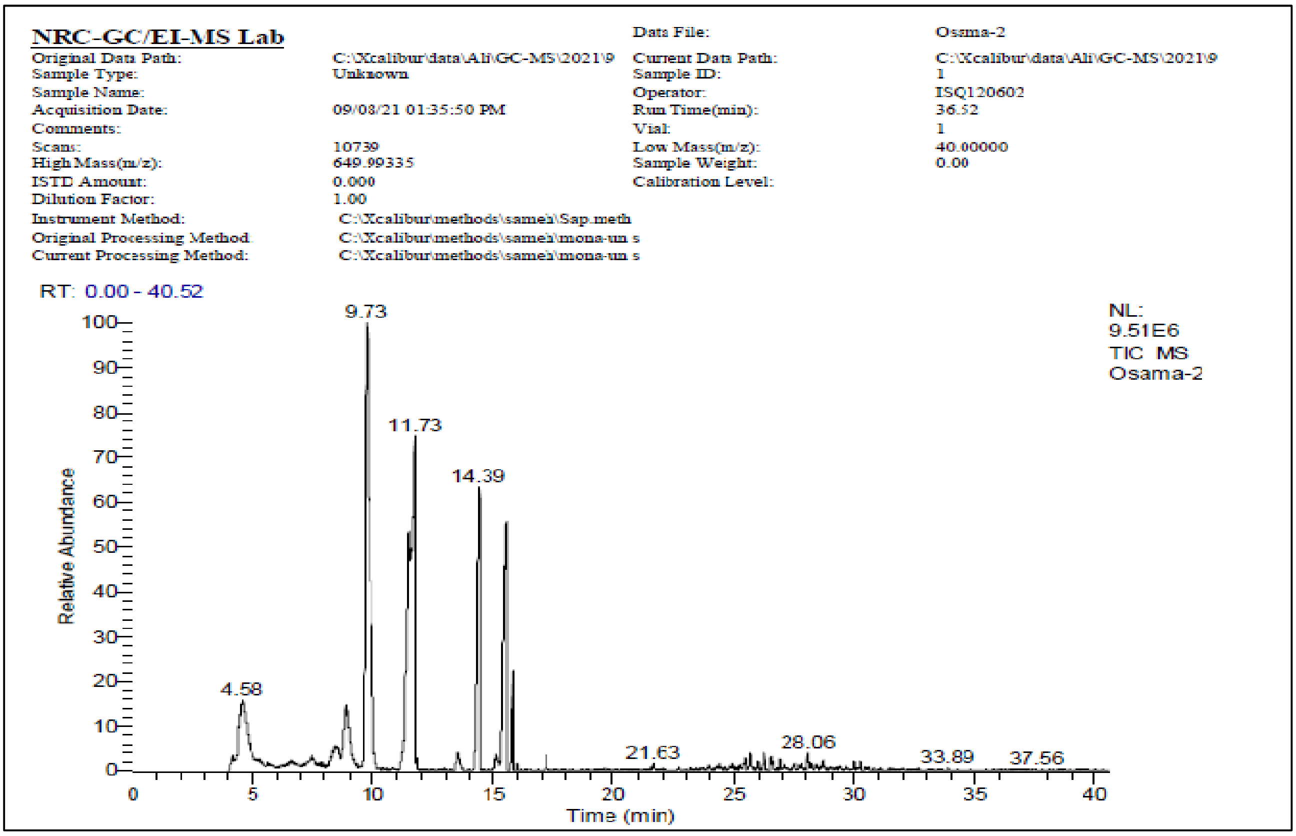

Figure S4: Monosaccharide compositions spectrum of *Zygophyllum album*

2. Amino acids compositions:

```
Acq. Operator     : SYSTEM
Sample Operator   : SYSTEM
Acq. Instrument   : hplc-1                         Location : Vial 1
Injection Date    : 11/25/2021 3:05:44 PM
                                                 Inj Volume : Inj prog
Acq. Method       : C:\CHEM32\1\METHODS\AMINO ACIDS 2021.M
Last changed      : 11/18/2021 4:18:40 PM by SYSTEM
Analysis Method   : C:\CHEM32\1\METHODS\RAPAMYCIN.M
Last changed      : 12/20/2021 3:58:32 PM by SYSTEM
                    (modified after loading)
Additional Info   : Peak(s) manually integrated
```

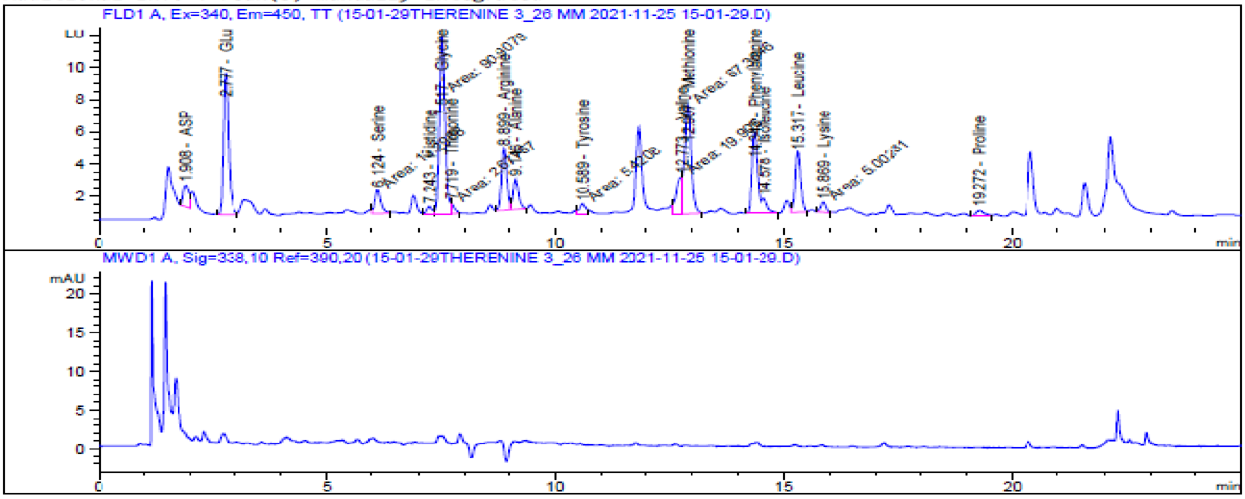

Figure S5: Amino acids compositions spectrum of *Anabasis ehrenbergii*

```
Acq. Operator     : SYSTEM
Sample Operator   : SYSTEM
Acq. Instrument   : hplc-1                         Location : Vial 1
Injection Date    : 11/25/2021 2:35:26 PM
                                                 Inj Volume : Inj prog
Acq. Method       : C:\CHEM32\1\METHODS\AMINO ACIDS 2021.M
Last changed      : 11/18/2021 4:18:40 PM by SYSTEM
Analysis Method   : C:\CHEM32\1\METHODS\RAPAMYCIN.M
Last changed      : 12/20/2021 3:58:32 PM by SYSTEM
                    (modified after loading)
Additional Info   : Peak(s) manually integrated
```

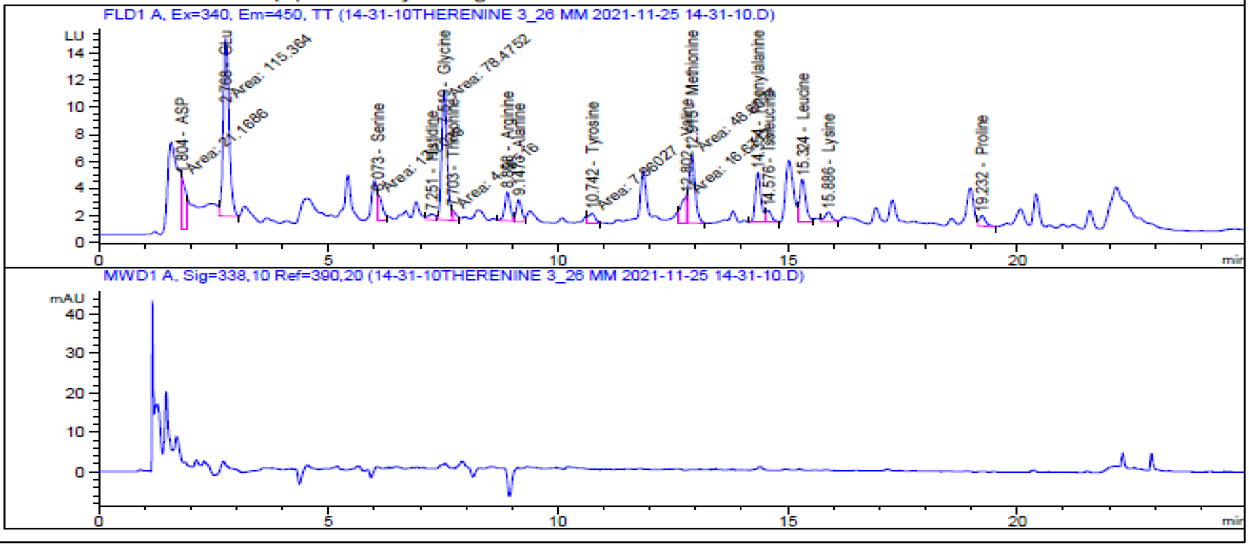

Figure S6: Amino acids compositions spectrum of *Suaeda aegyptiaca*

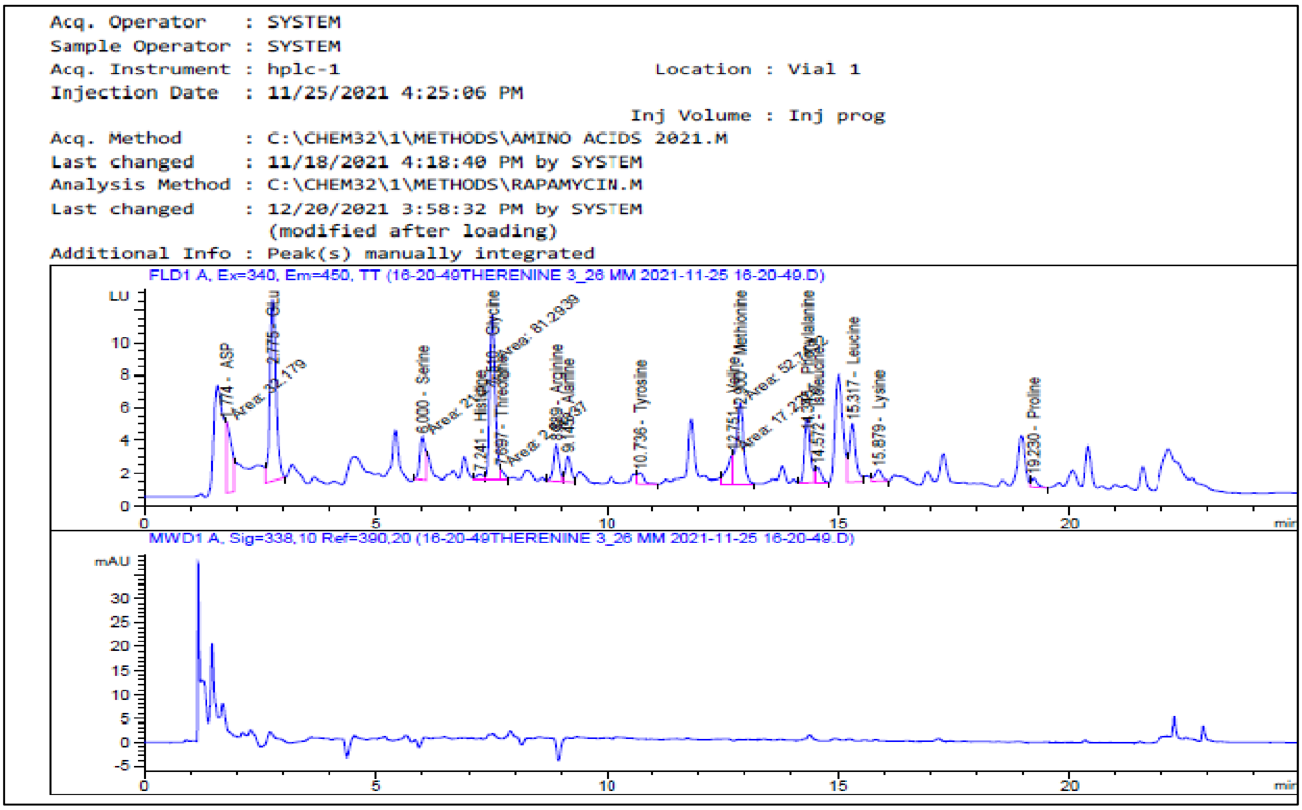

Figure S7: Amino acids compositions spectrum of *Saueda monoica*

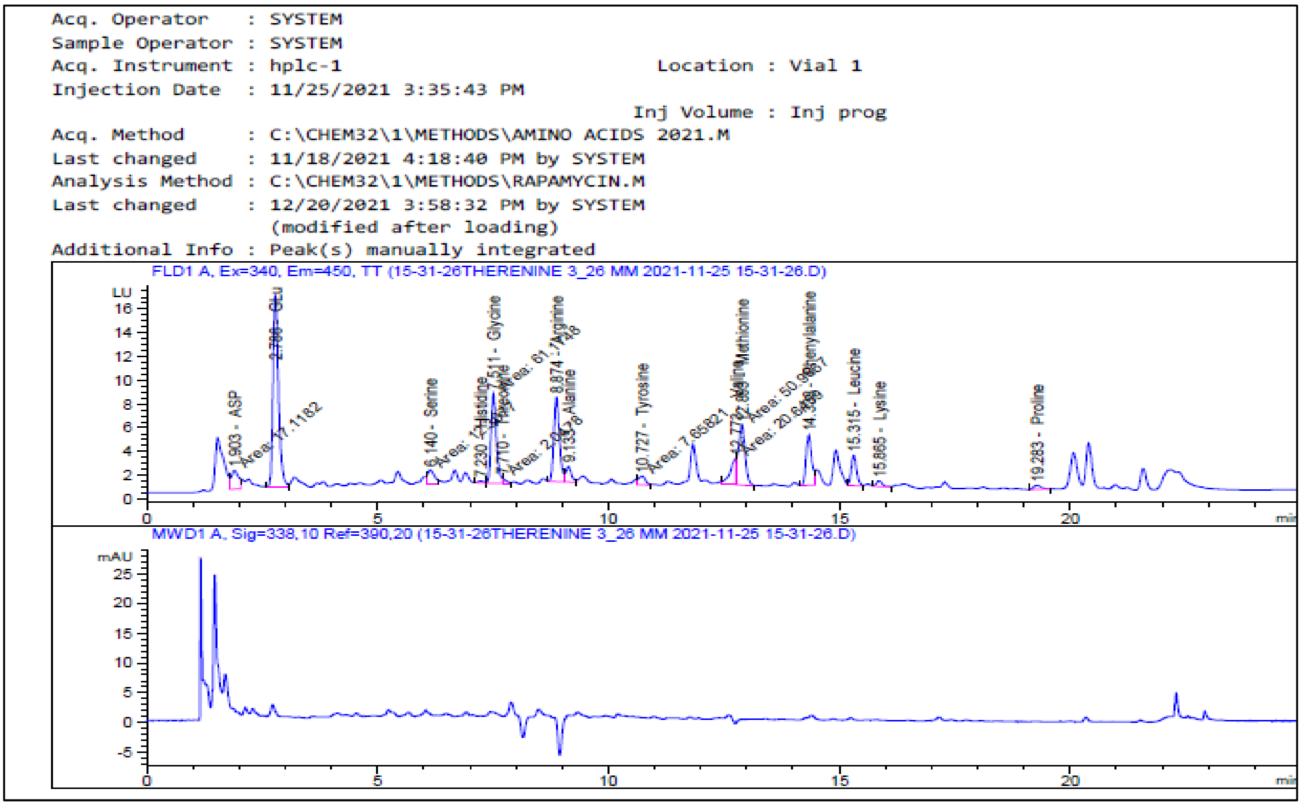

Figure S8: Amino acids compositions spectrum of *Zygophyllum album*

3. Polyphenol compositions:

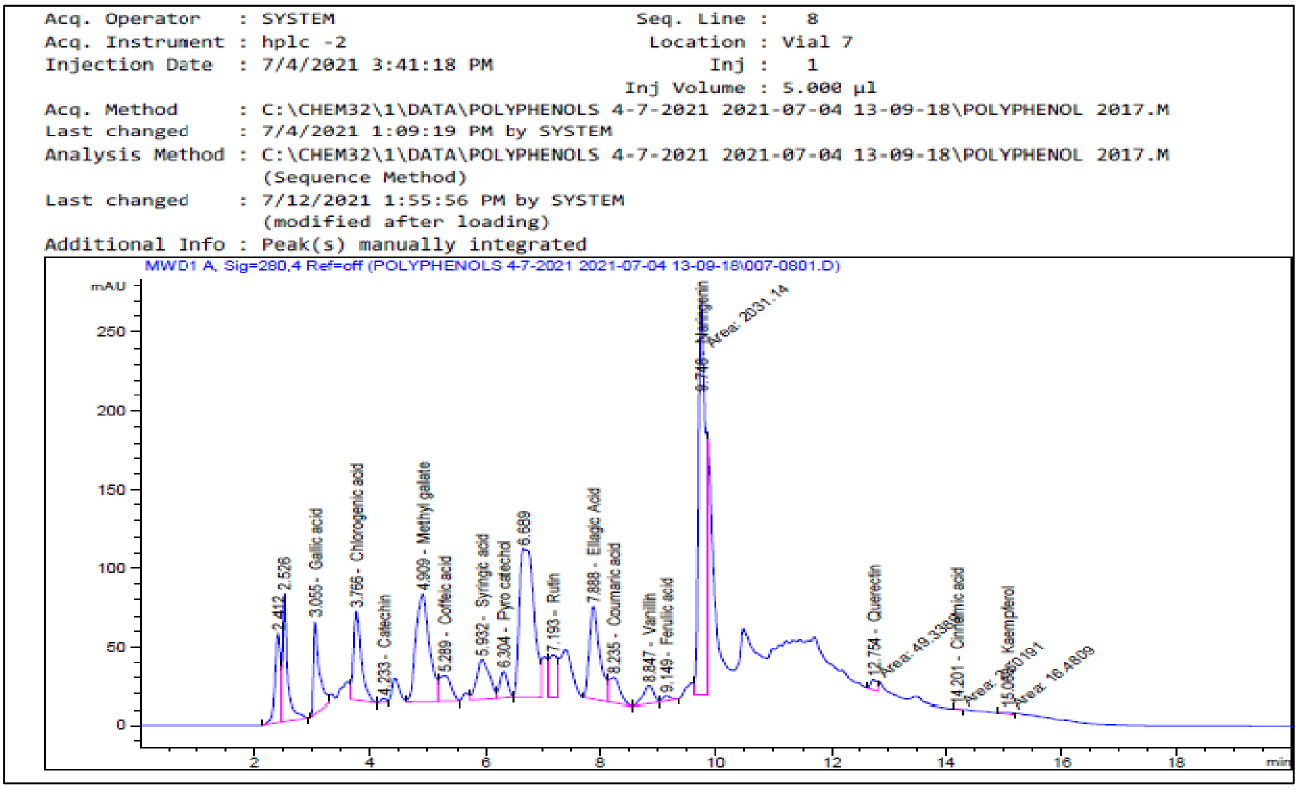

Figure S9: Phenolic compositions spectrum of *Anabasis ehrenbergii*

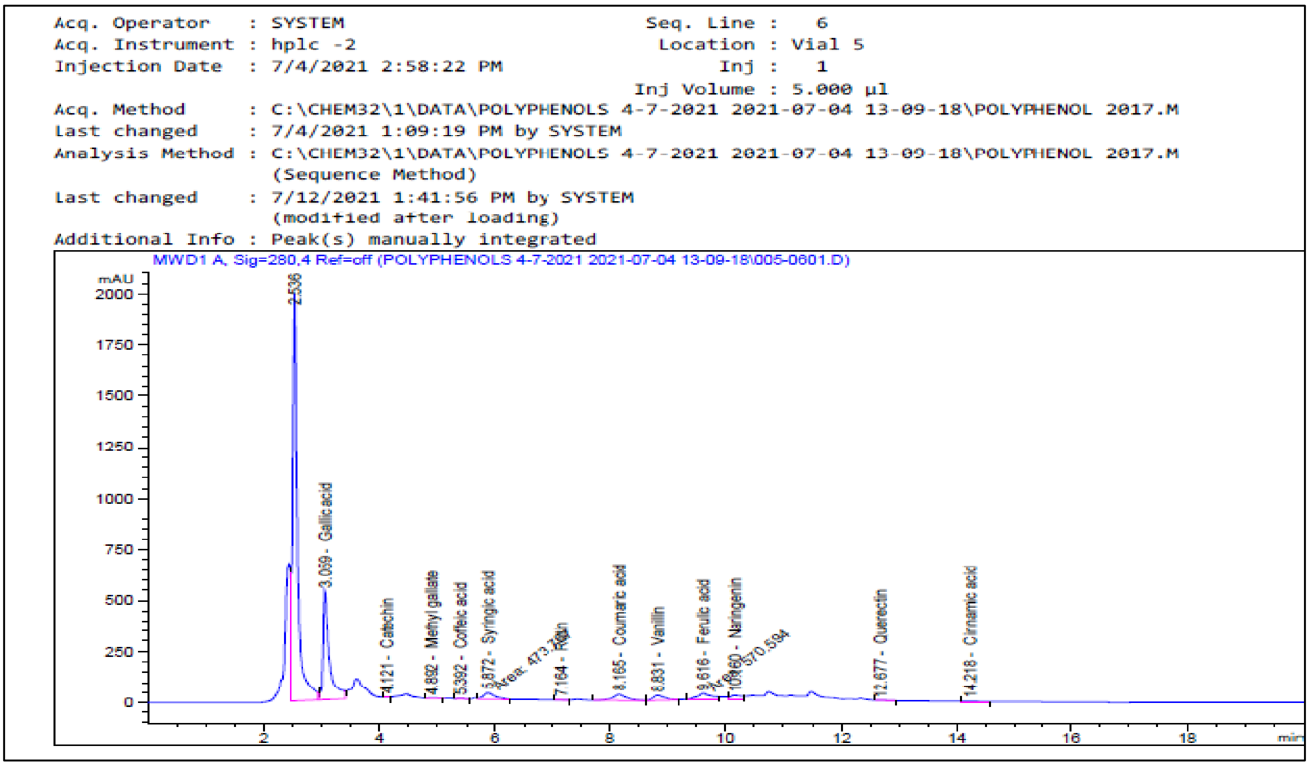

Figure S10: Phenolic compositions spectrum of *Suaeda aegyptiaca*

```
Acq. Operator    : SYSTEM                          Seq. Line :    9
Acq. Instrument  : hplc -2                          Location : Vial 8
Injection Date   : 7/4/2021 4:02:46 PM                   Inj :    1
                                                 Inj Volume : 5.000 µl
Acq. Method      : C:\CHEM32\1\DATA\POLYPHENOLS 4-7-2021 2021-07-04 13-09-18\POLYPHENOL 2017.M
Last changed     : 7/4/2021 1:09:19 PM by SYSTEM
Analysis Method  : C:\CHEM32\1\DATA\POLYPHENOLS 4-7-2021 2021-07-04 13-09-18\POLYPHENOL 2017.M
                   (Sequence Method)
Last changed     : 7/12/2021 1:55:56 PM by SYSTEM
                   (modified after loading)
Additional Info  : Peak(s) manually integrated
```

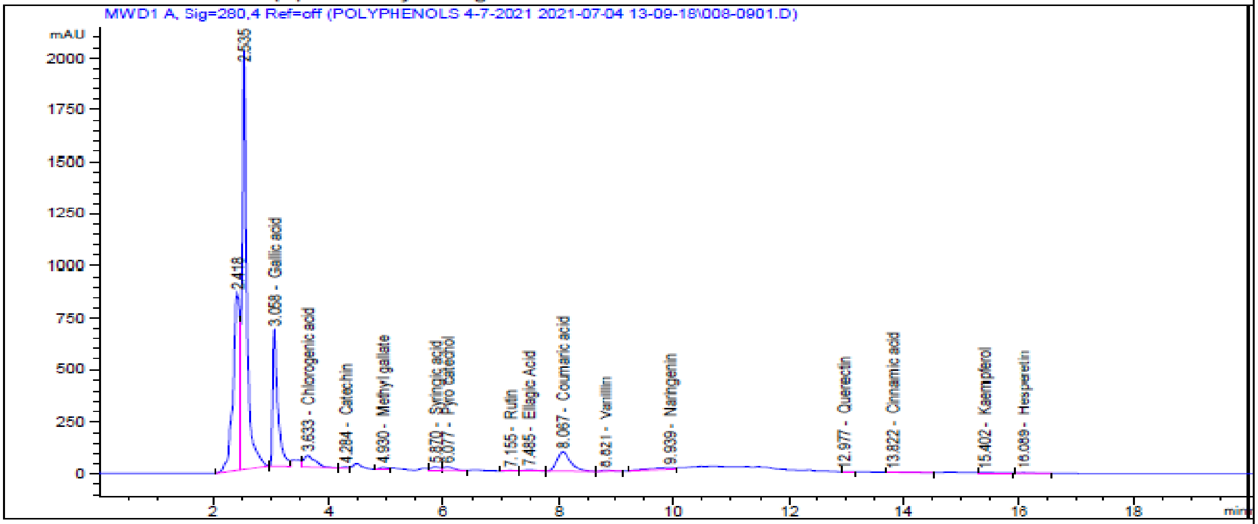

Figure S11: Phenolic compositions spectrum of *Saueda monoica*

```
Acq. Operator    : SYSTEM                          Seq. Line :    7
Acq. Instrument  : hplc -2                          Location : Vial 6
Injection Date   : 7/4/2021 3:19:50 PM                   Inj :    1
                                                 Inj Volume : 5.000 µl
Acq. Method      : C:\CHEM32\1\DATA\POLYPHENOLS 4-7-2021 2021-07-04 13-09-18\POLYPHENOL 2017.M
Last changed     : 7/4/2021 1:09:19 PM by SYSTEM
Analysis Method  : C:\CHEM32\1\DATA\POLYPHENOLS 4-7-2021 2021-07-04 13-09-18\POLYPHENOL 2017.M
                   (Sequence Method)
Last changed     : 7/12/2021 1:41:56 PM by SYSTEM
                   (modified after loading)
Additional Info  : Peak(s) manually integrated
```

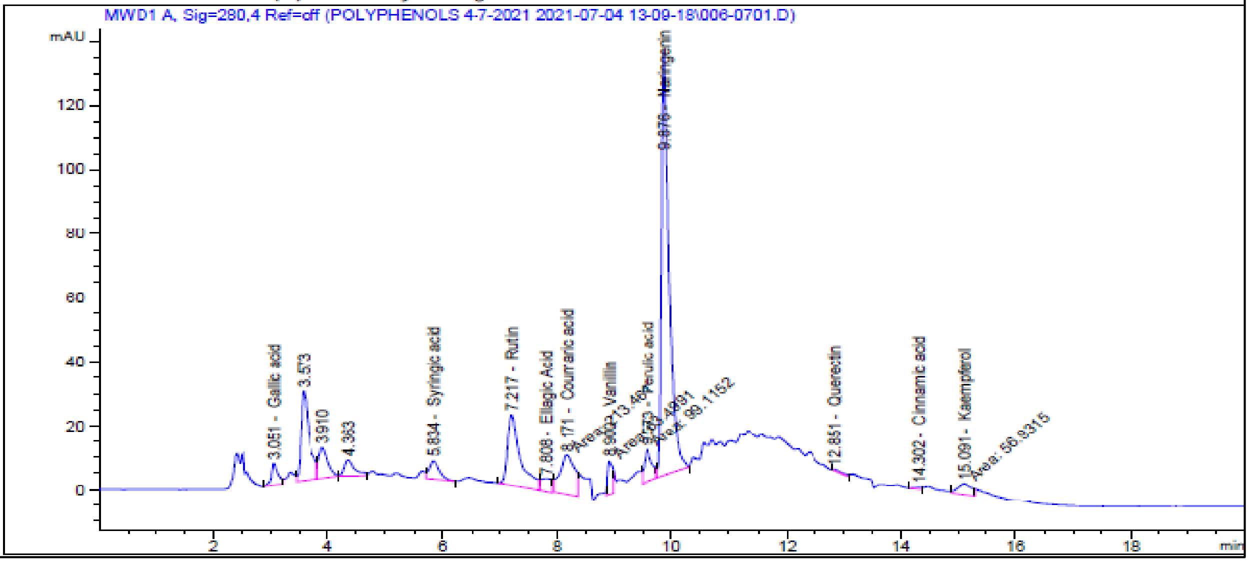

Figure S12: Phenolic compositions spectrum of *Zygophyllum album*

4. Fatty acids compositions:

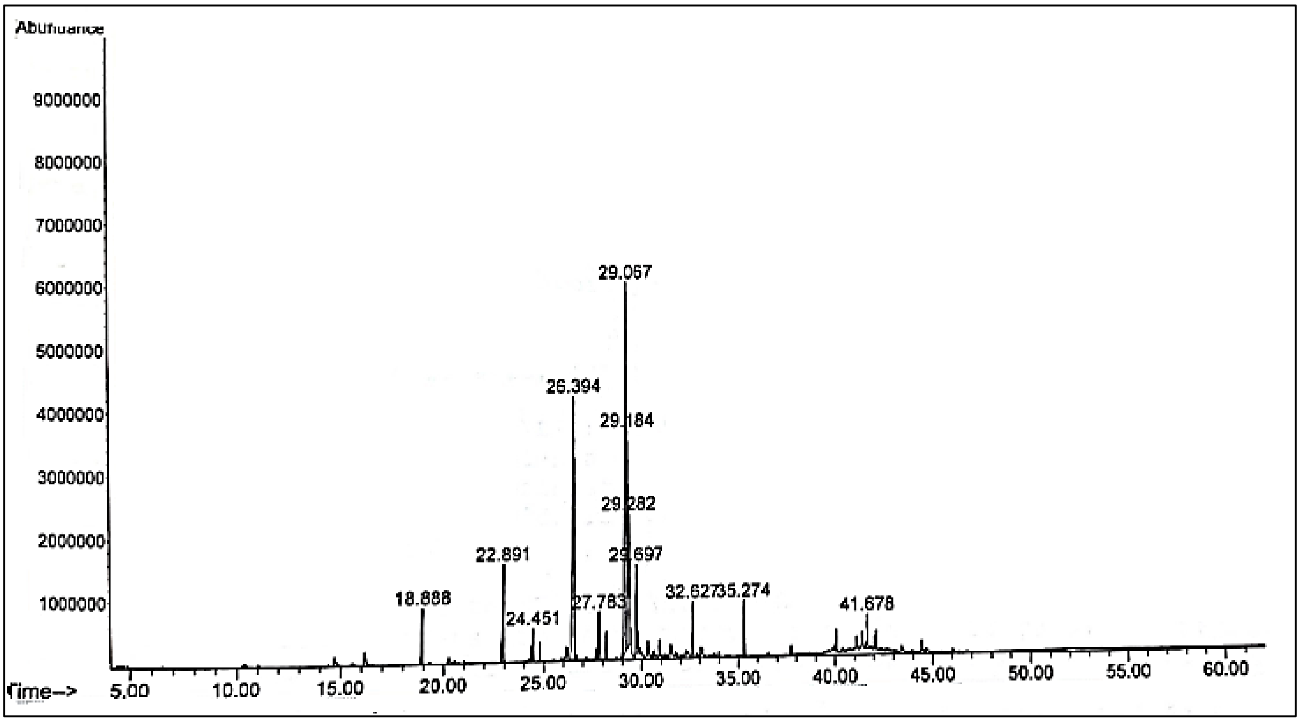

Figure S13: Fatty acids compositions spectrum of *Anabasis ehrenbergii*

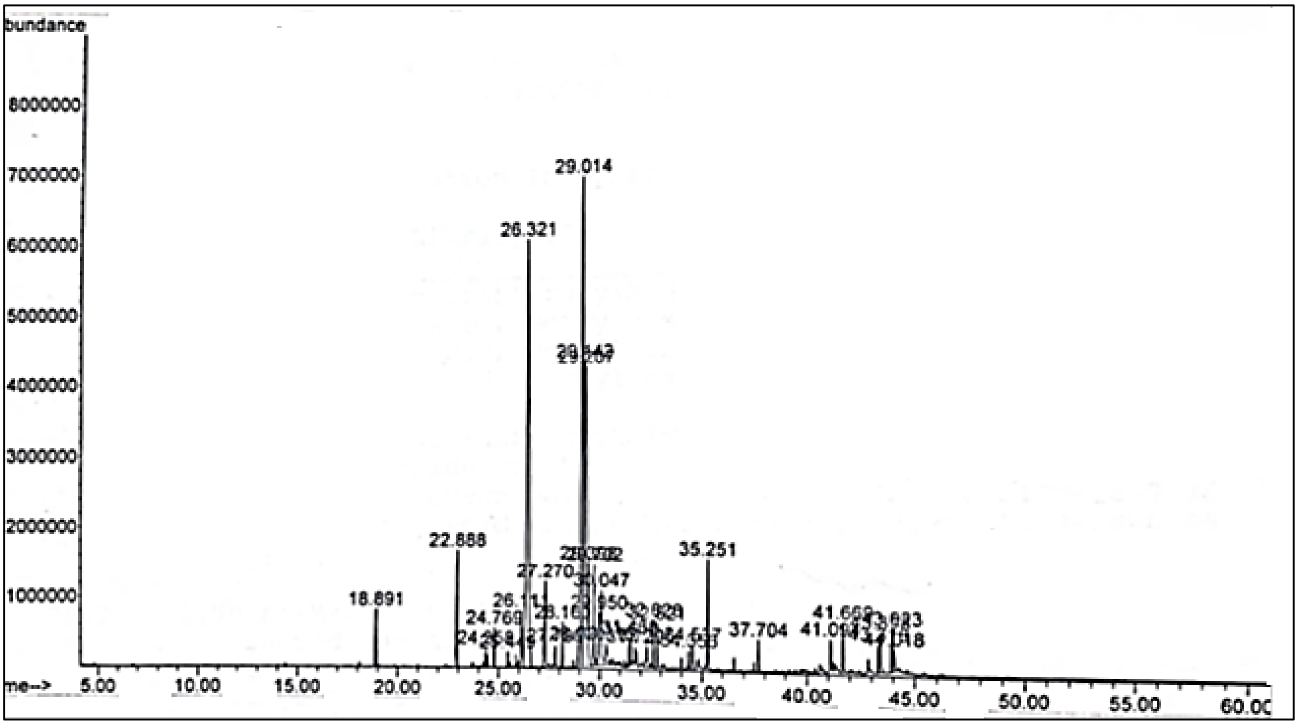

Figure S14: Fatty acids compositions spectrum of *Suaeda aegyptiaca*

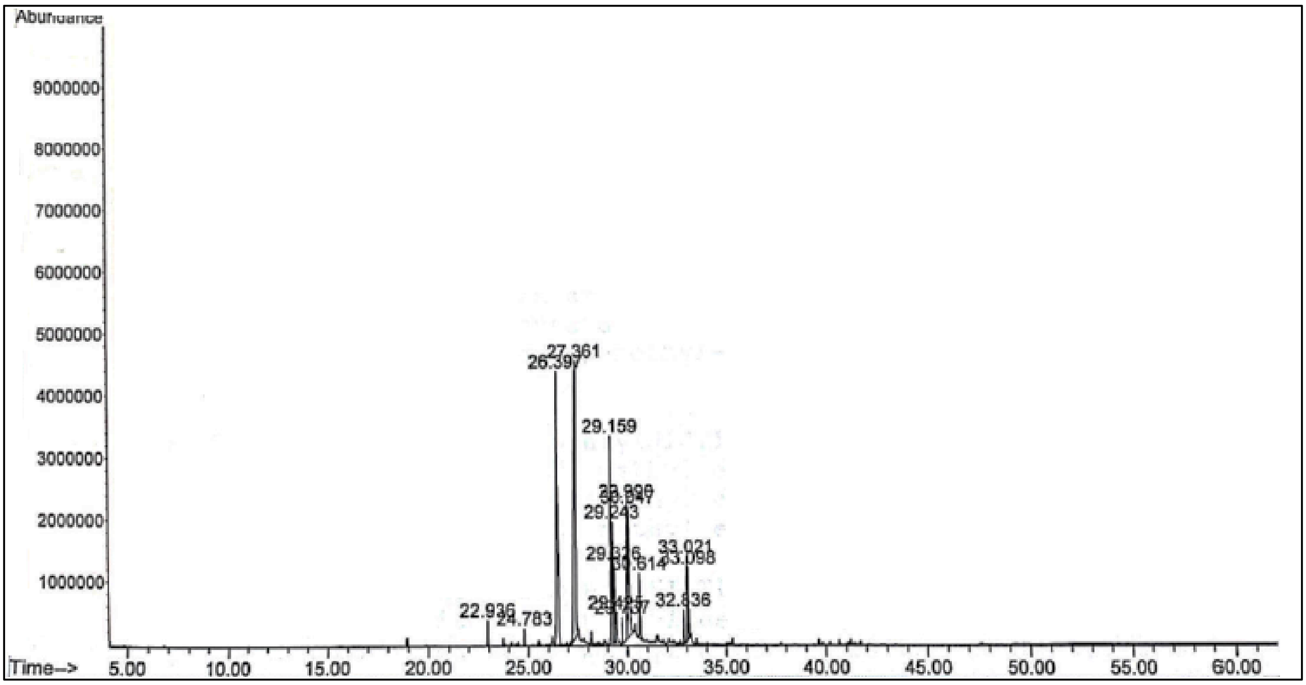

Figure S15: Fatty acids compositions spectrum of *Saueda monoica*

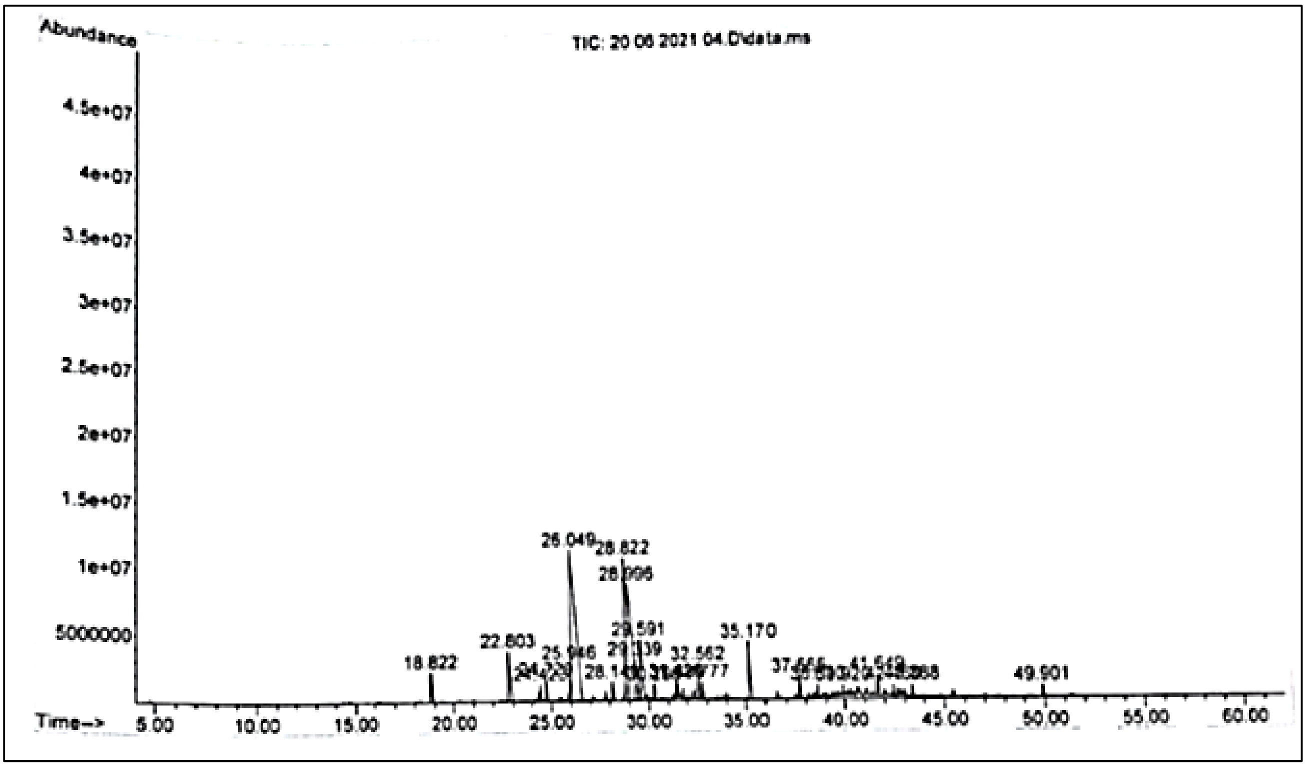

Figure S16: Fatty acids compositions spectrum of *Zygophyllum album*

5. Unsaponifiable matter analysis:

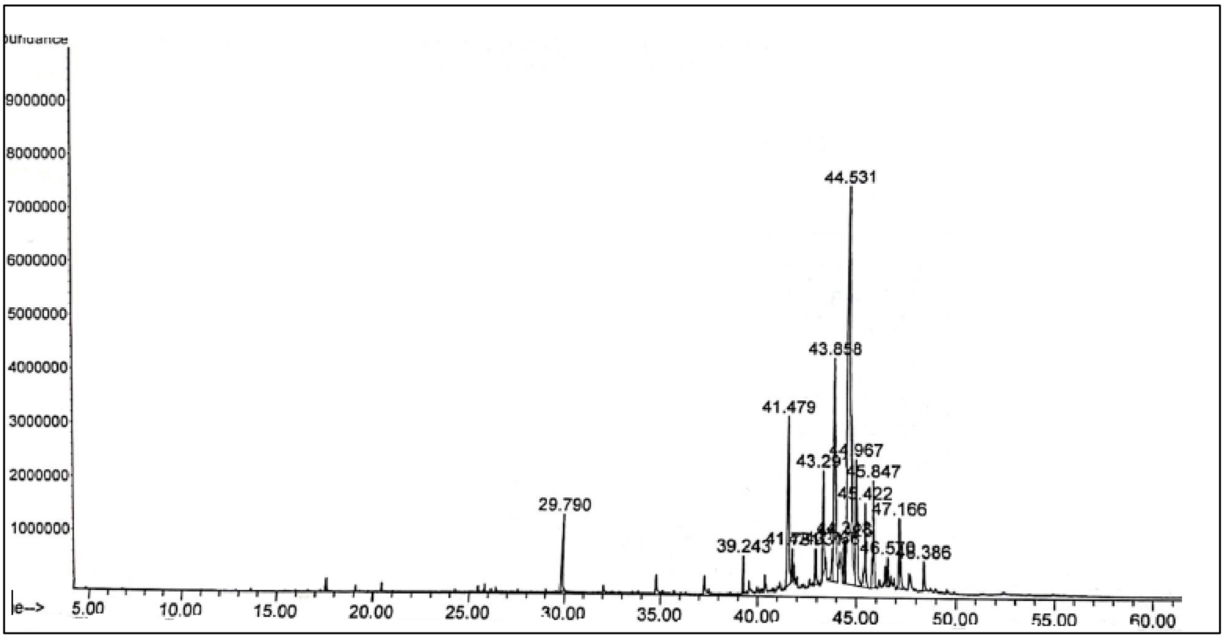

Figure S17: Unsaponifiable matter compositions spectrum of *Anabasis ehrenbergii*

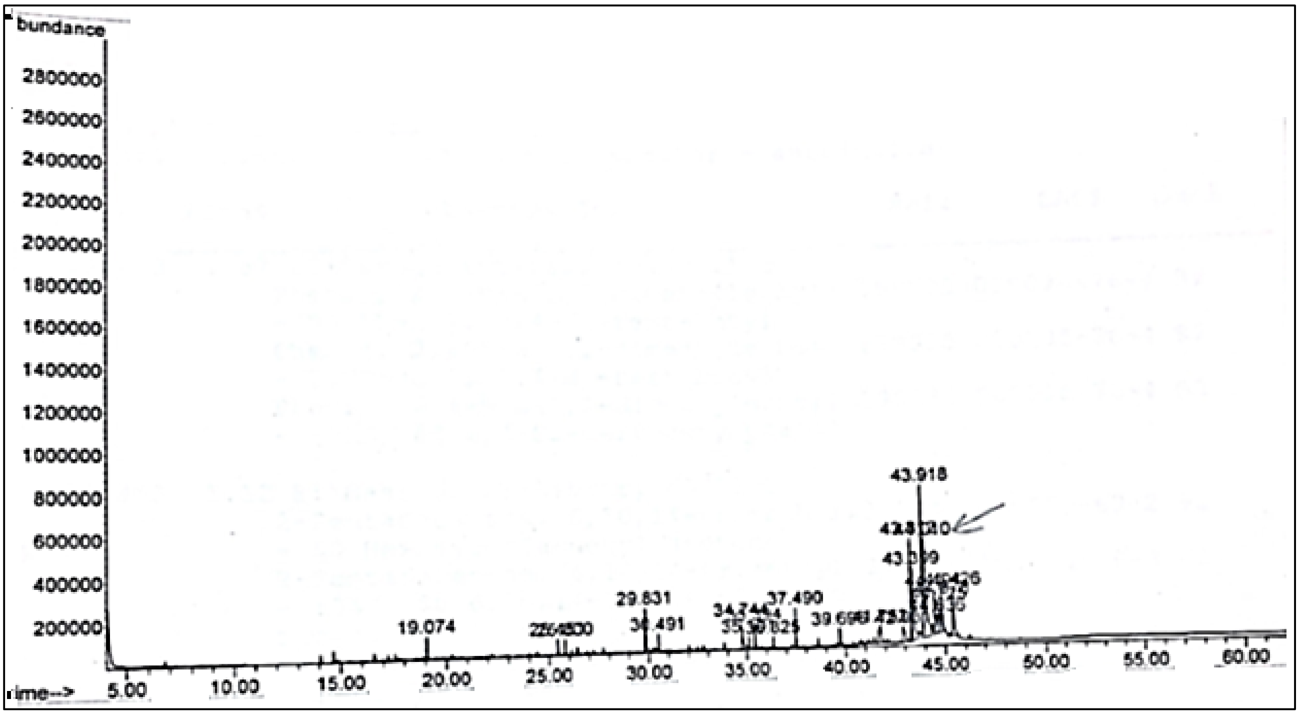

Figure S18: Unsaponifiable matter compositions spectrum of *Suaeda aegyptiaca*

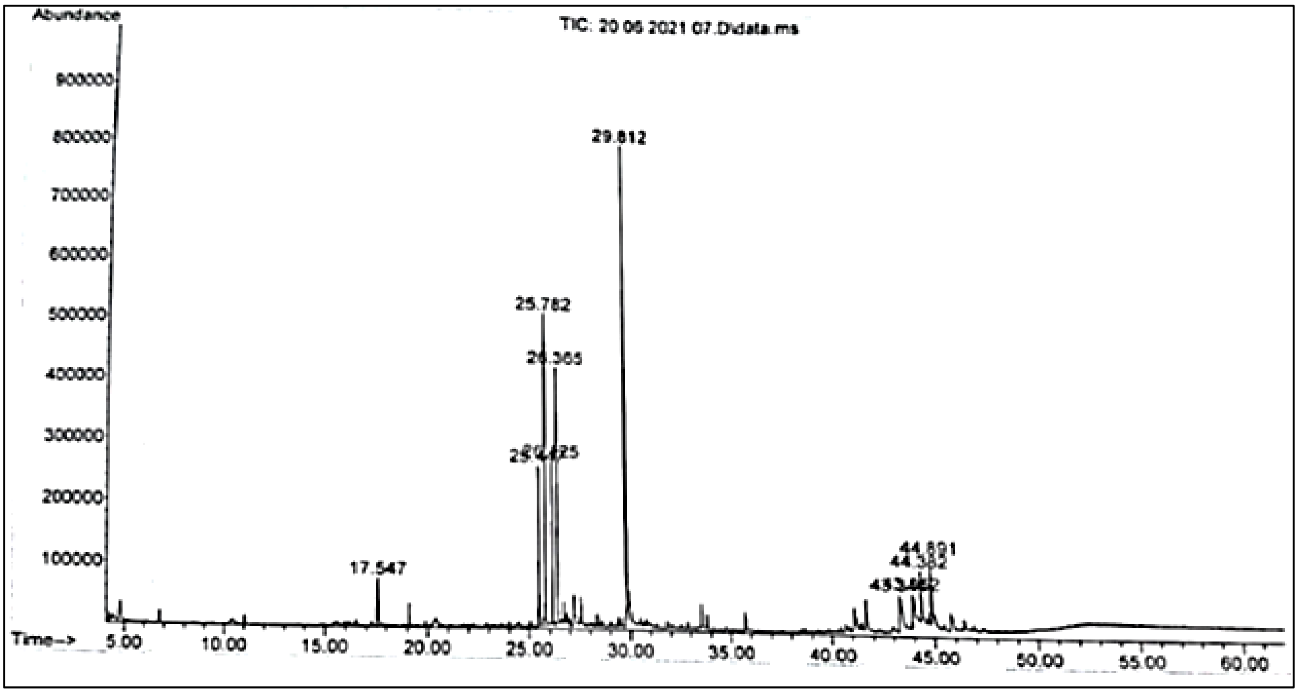

Figure S19: Unsaponifiable matter compositions spectrum of *Suaeda monoica*

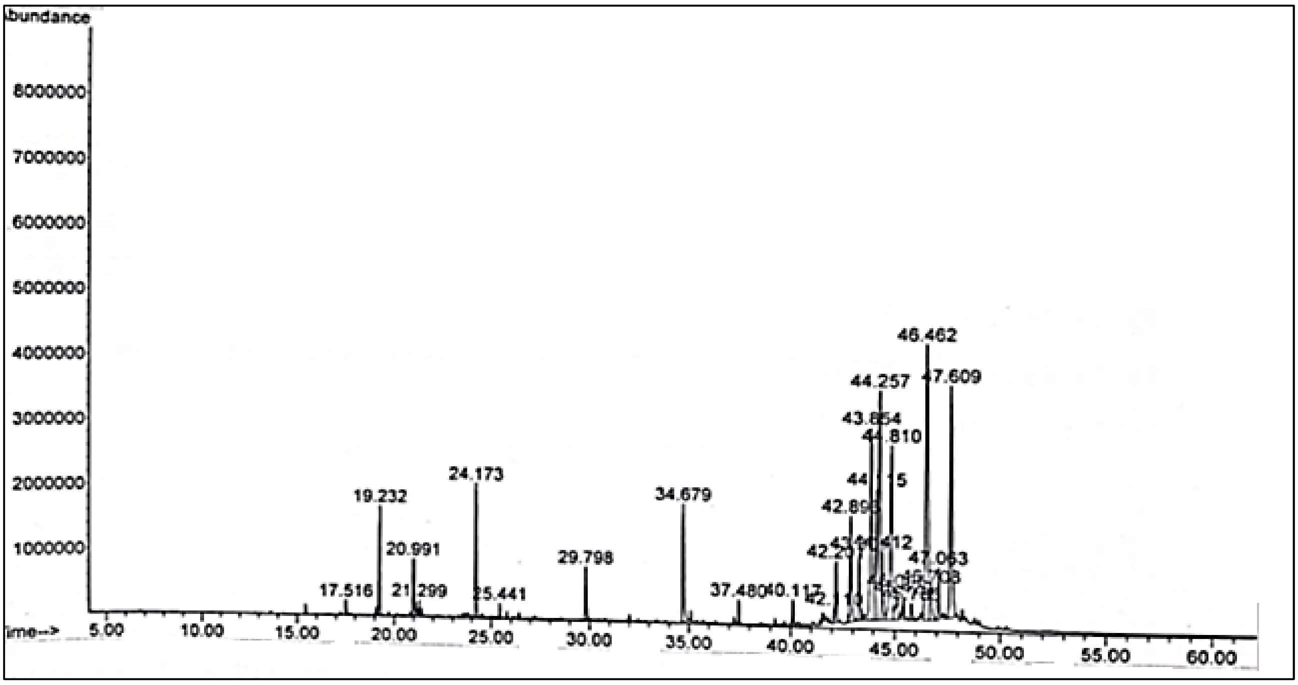

Figure S20: Unsaponifiable matter compositions spectrum of *Zygophyllum album*
